# Interevent-time distribution and aftershock frequency in non-stationary induced seismicity

**DOI:** 10.1038/s41598-021-82803-2

**Published:** 2021-02-11

**Authors:** Richard A. J. Post, Matthias A. J. Michels, Jean-Paul Ampuero, Thibault Candela, Peter A. Fokker, Jan-Diederik van Wees, Remco W. van der Hofstad, Edwin R. van den Heuvel

**Affiliations:** 1 Department of Mathematics and Computer Science, Eindhoven University of Technology, 5600 MB Eindhoven, The Netherlands; 2 Institute for Complex Molecular Systems, Eindhoven University of Technology, 5600 MB Eindhoven, The Netherlands; 3 Department of Applied Physics, Eindhoven University of Technology, 5600 MB Eindhoven, The Netherlands; 4 Université Côte d’Azur, IRD, CNRS, Observatoire de la Côte d’Azur, Géoazur, Nice, France; 5 Applied Geosciences, Netherlands Organisation for Applied Scientific Research (TNO), 3508 TA Utrecht, The Netherlands; 6 Department of Geosciences, Utrecht University, 3584 CB Utrecht, The Netherlands

**Keywords:** Statistics, Applied mathematics, Natural hazards, Seismology, Scaling laws

## Abstract

The initial footprint of an earthquake can be extended considerably by triggering of clustered aftershocks. Such earthquake–earthquake interactions have been studied extensively for data-rich, stationary natural seismicity. Induced seismicity, however, is intrinsically inhomogeneous in time and space and may have a limited catalog of events; this may hamper the distinction between human-induced background events and triggered aftershocks. Here we introduce a novel Gamma Accelerated-Failure-Time model for efficiently analyzing interevent-time distributions in such cases. It addresses the spatiotemporal variation and quantifies, per event, the probability of each event to have been triggered. Distentangling the obscuring aftershocks from the background events is a crucial step to better understand the causal relationship between operational parameters and non-stationary induced seismicity. Applied to the Groningen gas field in the North of the Netherlands, our model elucidates geological and operational drivers of seismicity and has been used to test for aftershock triggering. We find that the hazard rate in Groningen is indeed enhanced after each event and conclude that aftershock triggering cannot be ignored. In particular we find that the non-stationary interevent-time distribution is well described by our Gamma model. This model suggests that 27.0(± 8.5)% of the recorded events in the Groningen field can be attributed to triggering.

## Introduction

The damage caused by a single earthquake can extend far beyond its direct impact as a result of triggering additional aftershocks. Hence, the quantification of earthquake–earthquake interactions has been of prime importance among seismologists to assess time-dependent hazard. In induced-seismicity studies (e.g. related to water injection, fracking or gas production) it may be even more challenging to untangle the direct impact of varying human actions from delayed effects of transient aftershock sequences^1–4^; a sufficiently large data set may often not be available to model the full spatiotemporal process. Quantification of this triggering is crucial for moving towards a better understanding of the physical mechanisms governing induced seismicity. This research is a step towards distinguishing each earthquake as a background or triggered event.

Space–time clustering of natural earthquakes is traditionally captured in a self-exciting spatio-temporal point process, i.e., Hawkes process^5^. This process is defined via a hazard rate, the instantaneous event rate, partitioned into a background rate of events and a triggering function describing the interaction between events. The process can be simulated and fitted with the Epidemic-Type Aftershock-Sequence (ETAS) model^6–9^. It can describe spatial background variability and magnitude-dependent triggering, and is often used in practice. In steady state it contains besides the background rate up to four additional fit parameters, which together determine the fraction of triggered aftershocks^10^. As an alternative, a non-parametric probabilistic algorithm to quantify the background rate, assumed stationary, has been proposed^11^. Both methods facilitate the construction of declustered earthquake catalogs (i.e., with triggered events removed) by applying stochastic declustering. A graphical methodology based on space–time–magnitude nearest-neighbor distance between earthquakes has been introduced to establish the existence of two statistically distinct populations of background and triggered earthquakes^12^. The non-parametric and graphical methods rely on a constant expectation of earthquake–earthquake distances and aim at empirically obtaining information from the data without making additional assumptions on the underlying mechanism. For induced seismicity the facts (1) that the dynamic is non-stationary and (2) that the available data may be limited, make it extremely challenging to empirically estimate the hazard changes. ETAS-based methods that account for variations in seismicity (i.e. seismic swarms and induced seismicity) by estimating non-stationary background rates at time scales down to ten days are gaining attention^3,4,13–21^, e.g. by considering a sequence of time bins with piecewise constant backgrounds. The number of parameters then rapidly increases with the number of time bins. Nevertheless, background events and aftershocks have been accurately modeled in large induced-seismicity catalogs, containing up to thousands of events^13–16,18,20,21^. But such catalog sizes may not always be available, in which case the ETAS parameter estimates may become highly uncertain.

Triggering can also be studied from a survival-analysis point of view, where the focus is not on the cumulative number of events but on the interevent-time distribution. Stationary distributions for recurrence times of earthquakes on naturally active faults have been proposed based on the theory of self-organized criticality (SOC)^22,23^. It has then been argued that recurrence times on a single fault follow a Weibull distribution, while interevent times within a sufficiently large area are well approximated by an Exponential distribution: event times are then considered to be independent^24^. Joint modelling of fault-specific loading and unloading events is done via so-called renewal models^25^. In practice this may often be impossible due to the limited number of events and uncertainties in event location and event–fault association. In line with the SOC concept, scaling approaches have been successful in collapsing stationary interevent-time distributions of large seismicity data sets onto universal curves that suggest a (two-parameter) Gamma distribution^26–29^, but an ETAS-based model with four parameters has also been given^30,31^. Processes obeying stationary Gamma-like interevent-time distributions have also been studied in other phenomena, e.g. rock fracture and creep or shear in disordered materials^32–36^. Deviation from Exponential distributions has been presented as evidence for underlying physical correlations between events, but this has also been debated^37–39^.

An interevent-time distribution is uniquely defined by its hazard function, also known as the hazard rate^40^, for details see “Methods” and Supplemental text S1.1. For the Gamma distribution the hazard function at time *u* since the last event reads

1 $$\begin{aligned} h_{0} (u, \tau , k)=\frac{u^{k-1} \exp {(-u/ \tau )}}{\tau ^k \Gamma (k,u/\tau )}, \end{aligned}$$

where $\tau$ is the (time-)scale parameter, *k* is the shape parameter, and $\Gamma (k,u/\tau )$ is the upper incomplete Gamma function. The expected interevent time, being inversely proportional to the average event rate, equals $k\tau$. For $k=1$ the Gamma distribution simplifies to the memoryless Exponential distribution, which lacks event interactions and describes the interevent times of the background Poisson process, with average event rate $\tau ^{-1}$. For $k < 1$ the exponential factor in the probability-density function should in the asymptotic limit of very large *u* still be determined by the rate $\tau ^{-1}$ of the uncorrelated background events^41,42^. In real data a correction appears at finite but large *u* when high background rates give rise to strongly overlapping aftershock sequences and thus also to uncorrelated pairs of subsequent aftershocks. The correction is not rigorously exponential in *u* but may in practical data fitting easily be interpreted as such, which leads to overestimation of the background rate^28^. This effect applies to Gamma- as well as ETAS-based models. (Values $k > 1$ are unphysical since then the additional aftershocks would increase the average interevent time above the background Poisson value.) For stationary distributions the shape parameter *k* has indeed been recognized as the ratio of background-to-total events, with $\tau ^{-1}$ the background rate^41,42^.

**Figure 1:**
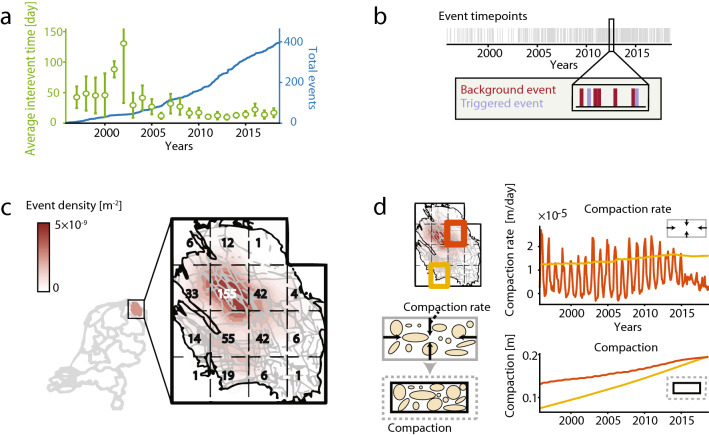
Groningen-field local characteristics and non-stationary induced earthquakes. (**a**) Yearly average interevent time (days) between two subsequent events in the field (green dots) and the evolution of the total number of events (blue line) from October 1995 until October 2018. (**b**) For each event (light grey) it is unknown whether this is a background (red) or a triggered event (purple). (**c**) Schematic overview of the faults (solid grey) in the field and the probability-density map ($\text {m}^{-2}$) of the induced-earthquake locations in the period 1995–2018 ($M\ge 1.3$, $n=397$). The field is divided into 15 grid cells and the total number of events per cell is presented. (**d**) The average compaction rate (m/day) and total compaction (m) of the ground soil over time for the two highlighted regions. For the red region the swing in compaction rate is due to the seasonal swing in gas production.

Gas production in the Groningen field in the North of the Netherlands has resulted in induced seismicity with a rapidly increased non-stationary activity, amounting to a catalog of 397 events above the magnitude of completeness $M = 1.3$ in the period 1995–2018 (Fig. 1a)^43–51^. The gas withdrawal causes reservoir compaction and associated stress build-up along pre-existing faults. When the stress reaches a threshold value, faults may be activated, induced earthquakes start to nucleate and seismic energy is released^2^. The development of the reservoir compaction (see Fig. 1d) causes the non-stationarity and hinders the use of the conventional statistical or probabilistic methods typically developed for stationary cases. There is no clear consensus on the importance of earthquake–earthquake interaction for Groningen’s induced seismicity^44,47,49,50^. Uncorrelated human-induced triggers of seismic activity may, via earthquake–earthquake interaction, each entail internally correlated bursts of duration longer than the minimal time resolution. The times between these triggers will be Poissonian distributed. The unavoidable thresholding of the catalog by a minimal magnitude, and the time resolution, will split individual bursts into separately observed events of minimal duration. This creates a catalog of such events including correlated aftershocks and with short interevent times that modify the Poisson distribution, while hiding silent earthquake excursions below the threshold^37,39,52^. To decluster such a limited and thresholded catalog and stochastically identify the original uncorrelated operational triggers will be the purpose of this paper, with Groningen as a case study. This requires generalizing the conventional statistical methods and simultaneously addressing the clearly visible spatial heterogeneity in activity and fault density over the field (Fig. 1c). In the case of substantial earthquake–earthquake interaction, the approach should be able to quantify the likeliness for each event to have been triggered by another event (Fig. 1b). It is only after answering this question that the direct effect of human action on induced seismicity can be faithfully quantified.

## Results

### Temporal non-stationarity

Modeling non-stationary seismicity brings complications far beyond those of steady state. With sufficient data these have already been addressed successfully by ETAS models^14–16,18,20,21^. But since our purpose is to consider the added complication of small catalogs we therefore choose as an alternative the Gamma form (1). It is admittedly more empirical, but it has a minimum number of parameters and contains the Poisson process for $k = 1$. Gamma-type data collapse has been observed in interevent statistics of steady-state cases, see e.g.^26,29,42^, although deviations may occur at the shortest interevent times^27,28,30,31^. As a non-stationary extension we propose a Gamma Instantaneous Accelerated-Failure-Time (IAFT) model. In this case the hazard for $k=1$ still equals $1/\tau (t)$, independent of the time *u* since a previous event, giving rise to a non-homogeneous Poisson process. We assume that case to represent the memoryless process of the operationally induced background events, with rate $1/\tau (t)$, while $k<1$ decreases the expected interevent time and thus indicates the presence of aftershocks. There is no unique modification of the Gamma distribution to account for non-stationarities and accurately model the process for $k<1$. To define our modified hazard, we choose to start from the process-controlled background rate and keep the functional form (1). We allow the background rate to depend on *J* time- and space-varying covariates $\varvec{y}= \left( y_{1},\dots ,y_{J}\right)$, e.g. injection/production rate. For the background rate in region *x* we introduce the functional relation

2 $$\begin{aligned} 1/\tau _{x}(t)=\exp \Big (\textstyle \sum _{j=1}^{J} \beta _{j}y_{j}(x,t)\Big )/\tau _{0}, \end{aligned}$$

where $y_{j}(x,t)$ equals the value of the $j{\text {th}}$ covariate at time *t* since the starting point, in region *x*, and $\beta _{j}$ represents the effect of the $j{\text {th}}$ covariate. We employ the exponential function to guarantee a positive background rate, and a linear first-order approximation of its argument. Note that this way of modeling the background rate does not depend on the choice for the Gamma distribution and could also be used in e.g. an ETAS model. When we assume that covariates are approximately constant in regions, the total background rate is the sum over the time-dependent background rates of all regions:

3 $$\begin{aligned} 1/\tau (t)=\textstyle \sum _{x\in X} 1/\tau _{x}(t), \end{aligned}$$

where *X* equals the field of interest. To distinguish between the global time *t* and interevent time *u*, let $t_{l}$ represent the point in time when the previous event occurred. Our Gamma IAFT model is then defined by linking the field hazard rate *h* at time $t=t_{l}+u$ to the total background rate at that time as:

4 $$\begin{aligned} h(u,t_{l},k)=h_{0}(u,\tau (t_{l}+u),k). \end{aligned}$$

In words, the Gamma IAFT hazard at time $t = t_{l} + u$ equals the hazard rate of a standard Gamma distribution with shape parameter *k*, but ‘accelerated’ with the time-scale parameter $\tau (t_{l} + u)$ of that instantaneous time. Note that *h* depends on the interevent time *u* via the functional form of $h_{0}(u,\tau ,k)$, as well as via a change in the global time $t=t_{l}+u$. Since the scale parameter $\tau (t)$ does change over the global time scale, the process is no longer renewal, in the sense that the interevent times remain not identically distributed^53^.

The earthquake process follows the hazard rate (4) and we assume that the background process is Exponential with hazard rate $1/\tau (t)$. The hazard can then be partitioned in the background rate $1/\tau (t_{l}+u)$ and the additional hazard due to triggering, $h(u,t_{l},k)-1/\tau (t_{l}+u)$. An event that occurs at time $t=t_{l}+u$ is thus triggered with probability

5 $$\begin{aligned} p_\text {triggered}(u, t_{l},k) = \frac{h(u,t_{l},k)-1/\tau (t_{l}+u)}{h(u,t_{l},k)}, \end{aligned}$$

for a formal proof see Supplemental text S4.1. Thus, interevent times giving rise to a substantial difference $h(u,t_{l},k)-1/\tau (t_{l}+u)$ are likely the result of earthquake–earthquake interaction. The triggering probabilities (5) will be used to estimate which fraction of the past events are aftershocks.

If the covariates, and thus $\tau (t_{l}+u)$, are approximately constant during an interevent period, the interevent-time distribution is well approximated by a Gamma distribution and *k* can be interpreted as the fraction of background events. If however the covariates do change rapidly, the interevent-time distribution is more complex. The fraction of background events then deviates from *k*, but it can still be derived from the sum of background probabilities (for details, see Supplemental text S5; this implies that our method of dealing with non-stationarity can, if necessary, also be applied to suitable more-parameter modifications of the Gamma distribution).

### Spatial non-stationarity

So far we have focused on the temporal non-stationarity of the hazard. Induced-seismicity studies often work with limited data, where most events are neighboring. The estimates of the Gamma IAFT model will therefore be improperly biased towards the behavior of active regions. Such spatial heterogeneity over the field should be incorporated in our hazard function to prevent overfitting. In the absence of interaction, i.e., the Exponential ($k=1$) case, the hazard for the field equals $1/\tau (t)$ and is by virtue of (3) uniquely partitioned in the local background rates. In the case $k<1$, the hazard (4) cannot be partitioned in such a way and weighting is needed to assign events to the different regions. A logical choice in line with (3) is to divide the total-field hazard through weights proportional to the local background rates:

6 $$\begin{aligned} h_{x}(u,t_{l},k)=\frac{1/\tau _{x}(t_{l}+u)}{1/\tau (t_{l}+u)}h(u,t_{l},k). \end{aligned}$$

This weighted approach will give due emphasis to the intensity in the less active regions and thus prevent overfitting of the seismically active regions. In practice, one can use regions within which the covariates $\varvec{y}$ vary somewhat to mitigate the impact of event-location uncertainty; so we use regional cumulative covariates instead (e.g. the total length of faults). The time-varying model defined by Eqs. (1)–(6), with pre-specified covariates, can be fitted to the data set of observed interevent times by maximizing the probabilistic likelihood. Information criteria^54^ can be used to decide between different models with different subsets of candidate covariates and to select a final model for the field under study.

The final model should be both temporally and spatially validated. The interpretation of the parameters and the estimation of the fraction of triggered events are only valid when the Gamma IAFT model fits the data of interest well.

### Case study: triggering in the Groningen gas field

We have partitioned the Groningen field into 15 equally sized regions, such that each cell contains a reasonable number of events (Fig. 1c). As candidate covariates we have considered the average compaction rate (m/day), the average cumulated compaction (m), the total length of faults (m) in the (*x*, *y*)-plane, the average fault dip (degrees), the fraction of faults with a strike angle between $45^{\circ }$ and $90^{\circ }$, the average fault throw (m) and the average throw-to-thickness (of reservoir) ratio per region. These covariates have been standardized for stability of the numerical optimization of the likelihood. Furthermore, we considered a critical total compaction by introducing a truncation cap, mimicking the compaction level at which all faults in a region have reached their fault strength. The final model has been selected by fitting on the 1995–2018 catalog.

As dominant covariates we identified the total compaction (*C*), the compaction rate $(\dot{C})$, the total fault length (*F*), the percentage of faults with a strike between $45^{\circ }$ and $90^{\circ }$ (*S*), the throw-to-thickness ratio (*R*) and the truncation cap (*c*), such that

7 $$\begin{aligned} 1/\tau _{x}(t)=\exp {\Big (\beta _{C}\min \{y_{C}(x,t), c\}+\textstyle \sum _{i\in \mathcal {Y}}\beta _{i}y_{i}(x,t)\Big )}/ \tau _{0}, \end{aligned}$$

where $\mathcal {Y}:=\{\dot{C}, F, S, R\}$. The shape-parameter estimate $\hat{k}$ is found to be 0.73 (0.031); the estimates of $\tau _{0}$, $\varvec{\beta }$ and of the cap variable *c* are given in Table 1. Note that in real catalogs, short interevent times cannot be detected because waveforms of successive events overlap in seismograms^55–57^. Parameter estimates obtained while ignoring this thresholding might therefore be biased. However, in the case study, with one minute as the shortest interevent time, a fit with a five minutes threshold does not deviate from the presented results. For details on the model fitting see Supplementary text S2.

**Table 1:** Gamma IAFT-model parameter estimates and corresponding standard errors for the Groningen case.

*k*	$\log (\tau _{0})$	$\beta _{\dot{C}}$	$\beta _{F}$	$\beta _{S}$	$\beta _{R}$	$\beta _{C}$	*c*
0.73 (0.03)	9.49 (0.97)	2.60 $\times 10^{4}$ (9.50 $\times 10^{3}$)	6.32 $\times 10^{-6}$ ($1.64\times 10^{-6}$)	3.44 (0.62)	-1.44 (0.42)	23.80 (0.84)	0.24 ($0.44 \times 10^{-2}$)

**Figure 2:**
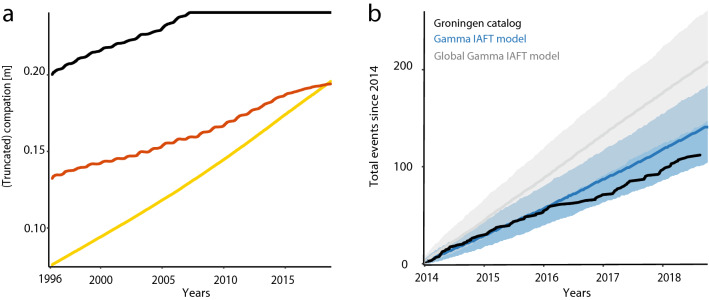
Results from the Gamma IAFT model for the Groningen case. (**a**) The evolution of the truncated total compaction (m) in the most active region (Loppersum, black, indicating critical stress from the end of 2007) compared to the compaction in the two regions highlighted in Fig. 1c. (**b**) Groningen catalog during the period 2014–2018 (black) and the median of thousand predicted catalogs using the (local) Gamma IAFT model (blue), respectively the global Gamma IAFT model that ignores spatial heterogeneity (grey). The $2.5\%$ and $97.5\%$ point-wise quantiles are presented as shaded areas (fit details in Supplemental text S3.3). Note that, after an offset in the first half of 2016, the real event *rate* still closely equals the median model prediction.

The estimated value of the cap variable indicates that all faults in the Loppersum region became critically stressed at the end of 2007, when the total compaction was 0.24 m in this most active region (Fig. 2a).

**Figure 3:**
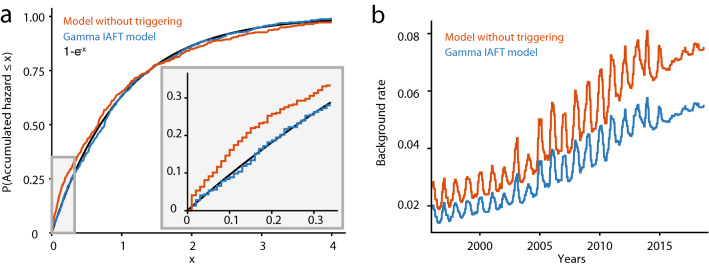
The effect of clustering in Groningen. (**a**) The left tail of the empirical distributions of the integrated hazard over each interevent period for the Exponential ($k=1$, red) and the Gamma (blue) IAFT models. These accumulated hazards should follow a unit Exponential distribution (black; Cox–Snell test^58^). The number of interevent times with a low accumulated hazard is overestimated in the $(k=1)$-model (inset: zoom), thus this model results in fewer short interevent times than observed in Groningen. (**b**) Background rate (events per day) based on the ML-estimates of the Exponential IAFT (red) and Gamma IAFT (blue) models.

Our non-stationary Gamma IAFT model proved to fit the multi-year seismicity rate and the temporal event clustering in the Groningen catalog very well. We have validated this by analyzing the distribution of the total accumulated (or integrated) hazard during the interevent period. It should follow a unit Exponential distribution when the true interevent-time distribution is used^58^, see Fig. 3a and “Discussion”. We can statistically accept that this is the case (Kolmogorov–Smirnov test, $p=0.68$). Furthermore, we did verify randomness of the model residuals over time (Wald–Wolfowitz runs test, $p= 0.16$), as well as the spatial (multinomial) event-count distribution over regions (Tailored $\chi ^{2}$-like test, $p=0.24$, for details see Supplemental text S3.2).

The sudden drop in gas production and associated compaction rate (Fig. 1d) in early 2014 can be used for further validation: if our model appropriately describes the causal mechanisms, we should be able to predict what happened after 2013 based on a model fitted on the 1995–2013 catalog only. And indeed the 2014–2018 catalog falls well within the uncertainty margins predicted by the 1995–2013 fit (Fig. 2b). This invariant prediction supports the causal interpretation of the estimates found in our study^59^. With that interpretation, the model can be used to analyze future hazards under hypothetical new production/compaction scenarios. For real seismicity prediction it is crucial that such drivers, as in this case compaction, can also be predicted accurately.

## Discussion

In this study we have introduced a novel Gamma IAFT model for analyzing interevent-time distributions and estimating the aftershock frequency that will be particularly useful in data-scarce induced-seismicity studies. For the Groningen gas field the estimated shape parameter $\hat{k}$ significantly differs from 1, from which we conclude the presence of clustering (Likelihood-ratio test, $p<0.0001$). Figure 3a analyzes the distribution of accumulated hazards and illustrates that the Exponential IAFT model underestimates the number of short interevent times and thus underestimates the temporal clustering of events. The misfit of the Exponential IAFT model can be overlooked when considering the fit to only the cumulative number of events, which obscures differences between models (see e.g. Fig. S7). The difference between the background rates estimated from the Gamma IAFT-model fit and the Exponential IAFT-model fit (Fig. 3b) highlights the importance of modelling clustering.

**Figure 4:**
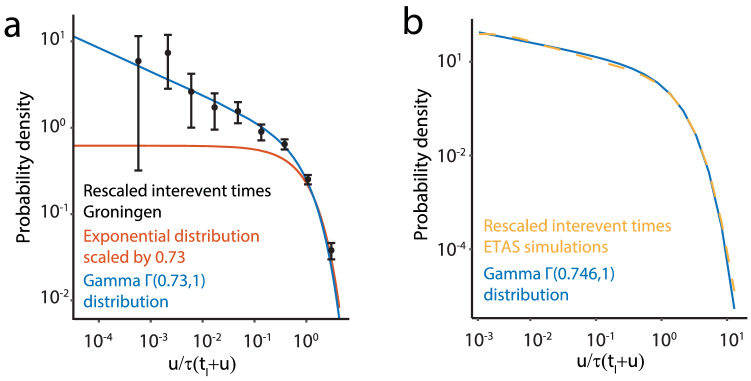
Gamma IAFT model compared to other models. (**a**) Estimated probability-density function (pdf) for the time-scaled interevent times, $u/\hat{\tau }(t_{l}+u)$, using a logarithmic binning of the 396 interevent times (black dots). For each estimate of the pdf a $95\%$ confidence interval is presented, for details see Supplemental text S3. Furthermore, the $\Gamma (\hat{k},1)$ pdf (blue) and the Exponential ($k=1$, red) pdf scaled by $\hat{k}$ are presented. (**b**) Distribution of interevent times from 500 Groningen-based synthetic ETAS catalogs, with a mean aftershock fraction equal to 0.766, scaled by the background rate at the time of events, $u/\tau (t_{l}+u)$ (dashed orange). A Gamma IAFT-model fit to all simulated interevent times follows the same distribution (blue).

In our Gamma IAFT model $\hat{k}$ does not a priori equal the fraction of background events. The fraction of triggered events can be estimated from the sum of triggered-event probabilities (5), for details see Supplemental text S4.2-3. For Groningen we find that 27.0% (95% CI 18.4%, 35.5%) of the past seismicity can be attributed to aftershocks. The Groningen case presents a clear separation between the time scales on which $\tau (t)$ and *h*(*u*) change as a function of their respective arguments, i.e., the background rate is approximately constant over the course of an interevent period. The interevent-time distribution is thus accurately approximated by the Gamma function and the estimated fraction of aftershocks indeed numerically equals $1-\hat{k}$. As a consequence of the scale separation, the temporal non-stationarity can be scaled out; the time-scaled data and the distribution $\Gamma (\hat{k},1)$ are given in Fig. 4a. Based on our model each event can be stochastically labeled using its probability (5) of being triggered, resulting in a stochastically declustered seismic catalog (see e.g. Fig. S13).

The risk of ignoring spatial heterogeneity, with results biased towards the faults in active regions, can also be shown. For this we fitted, instead of the local model (7), a model with global covariates $y(t)=\textstyle \sum _{x=1}^{15}y(x,t)$:

8 $$\begin{aligned} 1/\tau (t) = \exp \left( \beta _{C}\min \left( y_{C}(t), c\right) +\beta _{\dot{C}} y_{\dot{C}}(t)\right) /\tau _{0}, \end{aligned}$$

(the maximum-likelihood estimates for this model based on the catalog up to 2013 have been listed in Table S7). The uncertainty margins of the 2014–2018 catalog predicted from model (8) do not capture the real Groningen catalog (Fig. 2b). Modelling spatial non-stationarity is thus of major importance to obtain causal effect estimates of the operational parameters.

In this research we assumed that the shape parameter *k* was stationary. To validate this assumption we fitted the model for the periods 1995–2010 ($\hat{k}=0.775 (0.045)$, $n=180$) and 2011–2018 ($\hat{k}=0.715 (0.041)$, $n=217$). The $95 \%$ confidence interval for the change in shape parameter ($k_{\text {2011-2018}}-k_{\text {1995-2010}}$) equals $(-0.18,0.06)$. This sensitivity analysis shows that the difference is small compared to the variability in the data and could very likely occur assuming a constant *k*. Therefore we conclude that the increase in seismic activity in the Groningen field should be mainly attributed to the change in human-induced background events rather than to an increased earthquake-interaction mechanism.

Simulations with a Groningen-based temporal ETAS model indicate that the latter may still be in the class of models that give rise to approximately Gamma IAFT interevent-time distributions, as exemplified in Fig. 4b (for details see Supplementary text 5.1); the ETAS parameter estimates are highly uncertain here due to the small catalog. Moreover, in this example the parameter-poor Gamma fit to the synthetic ETAS catalogs proves more efficient than the ETAS fit to the synthetic ETAS catalogs. Here efficiency refers to the standard error of the estimated fraction of aftershocks, which is higher in the ETAS fit than in the Gamma fit (Brown–Forsythe test, $p<0.0001$). Thus when a limited data set is available, the Gamma IAFT model, which also naturally extends existing approaches of steady-state cases^26,29,41,42^, can be more useful to describe e.g. aftershock statistics because of the lower uncertainty in the parameter estimation.

As mentioned in previous sections, with a Gamma-model fit (as well as an ETAS-model fit) the Poisson background rate may be overestimated, due to overlapping aftershock sequences that introduce near-Poissonian interevent times, while deviations from Gamma-type behavior may occur at the shortest interevent times^27,28,30,31^. Our estimate of the fraction of aftershocks in the Groningen field heavily relies on the appropriateness of the Gamma IAFT-model fit, which was validated using the distribution of the accumulated hazard. To verify how sensitively this Cox–Snell test can detect deviations from a Gamma distribution, we simulated 397 events from hypothetical (stationary) ETAS models with different aftershock fractions, choosing parameters that enforce deviations from a Gamma distribution, for details see Supplemental text S5.2. For all settings, the distribution of the accumulated hazard under a Gamma model did deviate from the unit Exponential distribution in at least $79.0\%$ of the 500 simulations. This high statistical power does support our choice for the Gamma model, which gives that 27.0% (95% CI 18.4%, 35.5%) of the recorded events in the Groningen field can be attributed to earthquake–earthquake triggering.

Our novel statistical approach is suitable for small data sets and allows quantitative model validation. Goodness-of-fit tests, in particular to exclude significant deviations at very short interevent times, are a crucial part of the analysis. The Groningen case study illustrates the potential of the application of survival models in induced-seismicity studies. But their applicability is broader. The methodology is non-specific and can also be applied to other scenarios of induced seismicity or other hazards with spatiotemporal non-stationarities when data on the hazard drivers are available (e.g. data on climate factors that induce spreading forest fires).

For small data sets parsimonious multivariate models should ideally be developed as well, to address the joint distribution of interevent times, distances and magnitudes^60^, which is ignored in our current approach. This should complement spatio-temporal ETAS models that can effectively be used when enough data are available. To that end the hazard of the IAFT model could be extended by including magnitudes of past events and distances with respect to previous event locations. In such future research the trade-off between model complexity and variability of the estimates should be central.

Our study can help to develop further insight in the relative importance between human-induced operational forcing and triggered effects when data are scarce. Such insights will be crucial to develop safe clean-energy solutions for our planet.

## Methods

Methods are elaborately described in the Supplemental Information. Below, the main aspects of concepts, methods and data are highlighted.

### Hazard function

In modeling the distribution of a random interevent time *U*, the central concept is the hazard rate, equal to

9 $$\begin{aligned} h(u) = \lim _{\epsilon \rightarrow 0} \frac{\mathbb {P}(U\le u+\epsilon |U>u)}{\epsilon } = -\frac{d \log (S(u))}{du}, \end{aligned}$$

where $S(u)=\mathbb {P}(U>u) = 1-F(u)$ is the survival function. In statistical seismology the instantaneous event rate or hazard rate is often referred to as the intensity function. $H(u)=\int _{s=0}^{u} h(s) ds$ is the integrated (or accumulated) hazard, and $f(u)=-\frac{d S(u)}{du}$ represents the probability-density function (pdf). Then,

10 $$\begin{aligned} S(u)&= \exp (-H(u)). \end{aligned}$$

### Gamma Instantaneous Accelerated-Failure-Time model

In this research we model the hazard by a Gamma hazard that is ‘accelerated’ as a result of a time-varying scale parameter $\tau (t)$. Equation (4) can be expressed as

11 $$\begin{aligned} h(u, t_{l},k)= \frac{\tau _{0}}{\tau (t_{l}+u)} h_{0}\left( \frac{\tau _{0}}{\tau (t_{l}+u)} u, \tau _{0},k\right) , \end{aligned}$$

for some baseline value $\tau _{0}$ and with function $h_{0}$ as defined in (1). If the scale parameter $\tau (t)$ would be invariant over time this model is an example of a (stationary) Accelerated-Failure-Time (AFT) model^40^. Since this is not the case in our study, the relation does differ at every instance of time, for which reason we refer to this new model as an Instantaneous Accelerated-Failure-Time (IAFT) model. More details on the Gamma IAFT model and details on parameter estimation are presented in Supplemental text S1. The Gamma IAFT model is used to derive for each event the probability (5) of being a triggered event, as elaborated on in Supplemental text S4.

### Data

In the Groningen case study we have used the publicly available earthquake catalog from October 1995 until October 2018 provided by the Royal Netherlands Meteorological Institute (KNMI). Events with a magnitude lower than 1.3 were excluded from the catalog^61^, leaving us with 396 interevent times (median of 11.0 days and interquartile range (IQR) of [3.23, 26.8]). The yearly average interevent time and the development of the total event number over the period of interest are presented in Fig. 1a and the spatial distribution of the events is shown in Fig. 1b. The stationary covariates, viz. total fault length (median $95 \times 10^{3}$ m and IQR $[54 \times 10^{3}, 11 \times 10^{4}]$), percentage of faults having a strike between 45 and 90 degrees (median 0.42 and IQR [0.36, 0.48]) and throw-to-thickness ratio (median 0.12 and IQR [0.08, 0.26]) are derived from the geological *top Rotliegend surface model* from the Nederlandse Aardolie Maatschappij (NAM), provided via the Netherlands Organisation for Applied Scientific Research (TNO)^62^, and computed per grid cell as shown in Fig. 1c. The local compaction rate (median $7.31 \times 10^{-6}$ m/day and IQR $[4.15 \times 10^{-6} ,1.08 \times 10^{-5}]$) and total compaction (median 0.15 m and IQR [0.12, 0.18]) are derived from the compaction model provided by Shell^63^, see Fig. 1d. Further details on the standardization of covariates is presented in Supplemental text S2.1.

### Model selection

To model the interevent times in the Groningen field we write the Gamma scale parameter as a function of (time-varying) local covariates. The candidate covariates are presented in Table S1. Maximum-likelihood estimates of the parameters for different models, based on subsets of these candidate covariates, are implemented using statistical software [R]^64^. Subsequently, Akaike’s information criteria (AIC) are used to select the best model while preventing overfitting. More details on the model selection can be found in Supplemental text S2. Details on the validation of the model are presented in Supplemental text S3.

### Simulation

To simulate past-event catalogs based on the final model we rely on the survival-analysis relation $F(u)=1-\exp {\left( -H(u)\right) }$. In general a random variable with cumulative distribution function (cdf) *F* is generated by drawing a realization of a Uniform [0, 1] random variable *V* and evaluating the inverse cdf at this *V*, $F^{-1}(V)$. Equivalently, one could evaluate the survival function, $S=1-F$, at the random *V*. Our Gamma IAFT model defines the hazard rate, the integrated hazard *H*(*u*), and thus the cdf. More details are presented in Supplemental texts S3 and S5.

## Supplementary Information

Supplementary material 1 (pdf 10249 KB)

## Data Availability

The data that support the findings of this study are available from the corresponding author upon reasonable request.
