# Supplementary material for: Interevent-time distribution and aftershock frequency in non-stationary induced seismicity
Source: Sci Rep. 2021 Feb 11;11:3540. doi: 10.1038/s41598-021-82803-2 (PMC7878511; doi:10.1038/s41598-021-82803-2)
Supplement: Supplementary file 1 — Supplementary material 1 (pdf 10249 KB) [file 41598_2021_82803_MOESM1_ESM.pdf]

Supplementary Information for

Interevent-time distribution and aftershock frequency in non-stationary induced seismicity

R.A.J. Post, M.A.J. Michels, J.-P. Ampuero, T. Candela, P.A. Fokker, J.-D. van Wees, R.W. van der Hofstad, E.R. van den Heuvel

E.R. van den Heuvel.
E-mail: e.r.v.d.heuvel@tue.nl

This PDF file includes:

Figs. S1 to S17
Tables S1 to S11
SI References

1. Gamma IAFT model details and maximum-likelihood equations

1.1. Notation and survival analysis. In this Supplementary Information we use the standard notation in probability theory; random variables are represented by capital letters (e.g. X), while their realizations are written as lower-case letters (e.g. x). A probability is denoted as $\mathbb{P}(\cdot)$, the expectation as $\mathbb{E}(\cdot)$ and H_0 represent a statistical null-hypothesis. Estimates of parameters will be indicated by a hat, $\hat{\cdot}$.

We will use (and shortly state) standard concepts from survival analysis. The mathematical proofs and definitions used are well described in several textbooks, e.g. (1), but will be mostly omitted here. In survival, or failure time, analysis the central concept is the hazard rate, the instantaneous event rate, equal to

$$h(u) = \lim_{\epsilon \to 0} \frac{\mathbb{P}(U \leq u + \epsilon \mid U > u)}{\epsilon} = -\frac{d \log(S(u))}{du}, \tag{1}$$

where $S(u) = \mathbb{P}(U > u) = 1 - F(u)$ is the survival function. In statistical seismology this instantaneous event rate is often referred to as the intensity function.

The following equivalence relations are of great importance. Let $H(u)$ equal $\int_{s=0}^{u} h(s)ds$, the integrated (or accumulated) hazard, and $f(u)$ represent the probability-density function (pdf, $f(u) = -\frac{dS(u)}{du}$), then

$$S(u) = \exp(-H(u)), \tag{2}$$

$$f(u) = h(u)\exp(-H(u)), \tag{3}$$

$$h(u) = \frac{f(u)}{S(u)}. \tag{4}$$

1.2. Gamma Instantaneous Accelerated-Failure-Time model. The pdf and survival function of a random variable U_0 that follows a Gamma(τ_0, k) distribution equal

$$f_0(u) = \frac{u^{k-1}\exp\left(-\frac{u}{\tau_0}\right)}{\Gamma(k)\tau_0^k}, \tag{5}$$

$$S_0(u) = \frac{\Gamma(k, \frac{u}{\tau_0})}{\Gamma(k)}. \tag{6}$$

where $\Gamma(k,s) = \int_s^\infty x^{k-1}\exp(-x)dx$ is the upper incomplete Gamma function. Equivalently, the hazard function equals

$$h_0(u) = \frac{u^{k-1}\exp\left(-\frac{u}{\tau_0}\right)}{\tau_0^k \Gamma\left(k, \frac{u}{\tau_0}\right)}. \tag{7}$$

Finally the integrated hazard function equals

$$H_0(u) = \log\left(\Gamma(k)\right) - \log\left(\Gamma(k, \frac{u}{\tau_0})\right). \tag{8}$$

Let t equal the process time starting at $t = 0$. In this research we model the hazard by a Gamma hazard changing over time as a result of a time-varying scale parameter $\tau(t)$. More specifically, to model the interevent times in the Groningen field we model the Gamma scale parameter as a function of (time-varying) local characteristics. The scale parameter represents the inverse of the background rate and equals

$$\tau(t) = \frac{\tau_0}{\frac{1}{15}\sum_{x=1}^{15}\exp\left(\sum_{j=1}^{J}\beta_j y_j(x,t)\right)}, \tag{9}$$

where J is the number of covariates in the model. For fitting the Groningen data using 15 regions we used a factor $\frac{1}{15}$, such that $\tau(t)$ for a homogeneous field would equal $\tau_0/\exp\left(\sum_{j=1}^{J}\beta_j y_j(t)\right)$. Since the background rate is non-constant during the interevent time (total compaction is updated monthly) the interevent-time distribution is not a (simple) Gamma distribution. Let t_l represent the time of the last event, then the hazard of the distribution of the next interevent time, U, equals

$$h(u) = \frac{\tau_0}{\tau(t_l + u)} h_0\left(\frac{\tau_0}{\tau(t_l + u)}u\right). \tag{10}$$

More specifically this relation exhibits the feature of a stationary Accelerated-Failure-Time (AFT) model. A time-varying AFT model is defined such that $U_0 = \int_0^U \tau_0/\tau(t_l + u)du$ (2). We therefore refer to our model as an Instantaneous Accelerated-Failure-Time (IAFT) model (1). This distribution matches the geophysical context since the compaction (as a result of the gas production) accelerates the system, resulting in short interevent times with a probability equal to much longer interevent times in the case of no gas extraction.

Since, assuming $\tau(t_l+u)>0$, $\lim_{u\to\infty} h(u)-1/\tau(t_l+u)=0$, the interpretation of $1/\tau(t)$ as the background rate is indeed appropriate for this time-varying model. The IAFT model introduced can also be fit for more-parameter distributions of which the long-time hazard equals a Poisson hazard, i.e. converges to a constant.

In the Groningen case study we have only access to the monthly compaction rate and therefore the scale parameter is constant per month. The background rate is thus a discontinuous function with jumps at the end of each month. Let $n(u+t_l)$ equal the number of mutually exclusive months at time u after t_l. In this context, let s_j equal the time in days, after $t=t_l$, at the end of month j, with the exceptions that $s_0=0$ and $s_{n(u+t_l)}=u$. Then,

$$\begin{aligned} H(u) &= \int_0^u h(s)ds \\ &= \sum_{j=1}^{n(u+t_l)} \log\left(\Gamma(k, \tfrac{s_{j-1}}{\tau(s_j+t_l)})\right) - \log\left(\Gamma(k, \tfrac{s_j}{\tau(s_j+t_l)})\right). \end{aligned} \tag{11}$$

The local occurrence probabilities, $\mathbb{P}$(event occurred in region x | event occurred at time t), equal

$$w(x,t) = \frac{\exp\left(\sum_{j=1}^{J} \beta_j y_j(x,t)\right)}{\sum_{x=1}^{15} \exp\left(\sum_{j=1}^{J} \beta_j y_j(x,t)\right)}. \tag{12}$$

The local (region specific) hazard at time t in region x is defined as $h_x(u,t_l)=w(x,t_l+u)h(u)$. Finally, the log-likelihood of the observed interevent times and event locations in the Groningen field equals*

$$\begin{aligned} ll((u_1,x_1),...,(u_n,x_n)) =& \log\left(f((u_1,x_1),...,(u_n,x_n))\right) \\ =& \sum_{i=1}^{n} -H(u_i) + \log(h(u_i,x_i)) \\ =& \sum_{i=1}^{n} \sum_{j=1}^{n(u+t_l)} -\left[\log\left(\Gamma(k, \tfrac{s_{j-1}}{\tau(s_j+t_{l,i})})\right) - \log\left(\Gamma(k, \tfrac{s_j}{\tau(s_j+t_{l,i})})\right)\right] \\ &+ \left(\log\left(w(x_i,u_i)\right) + \log\left(\frac{u_i^{k-1}\exp\left(-\frac{u_i}{\tau(t_{l,i}+u_i)}\right)}{\tau(t_{l,i}+u_i)^k \Gamma\left(k, \frac{u_i}{\tau(t_{l,i}+u_i)}\right)}\right)\right), \end{aligned} \tag{13}$$

where n is the total number of events, u_i, respectively x_i, are the interevent time and the region of the ith event and $t_{l,i}=\sum_{j=1}^{i-1} u_j$.

1.3. Maximum-likelihood estimates. The parameters $(k,\tau_0,\boldsymbol{\beta})$ of the Gamma IAFT model are estimated by maximizing the log-likelihood defined in Eq. (13). These maximum-likelihood estimates are hard to find numerically as a result of the existence of local maxima for our complex log-likelihood. If $\tau(t)$ is constant over the course of each interevent time, then the log-likelihood simplifies to

$$\begin{aligned} ll((u_1,x_1),...,(u_n,x_n)) =& \sum_{i=1}^{n} \log\left(w(x_i,t_{l,i}+u_i)\right) + k\log(\tau(t_{l,i}+u_i)^{-1}) + (k-1)\log(u_i) \\ &- u_i\tau(t_{l,i}+u_i)^{-1} - \log(\Gamma(k)). \end{aligned} \tag{14}$$

Now, the joint distribution is a member of the exponential family. Therefore, the log-likelihood is concave and a unique maximum exists, see e.g. Appendix A in (3). Finding the parameters such that the gradient of the log-likelihood equals zero gives rise to this unique maximum. The components of the gradients can be derived and are equal to

$$\frac{\partial ll((u_1,x_1),...,(u_n,x_n))}{\partial k} = \sum_{i=1}^{n} \log\left(u_i\tau(t_{l,i}+u_i)^{-1}\right) - \psi(k), \tag{15}$$

$$\frac{\partial ll((u_1,x_1),...,(u_n,x_n))}{\partial \tau_0} = \sum_{i=1}^{n} \left(\frac{k}{\tau(t_{l,i}+u_i)^{-1}} - t_i\right)\tau(t_{l,i}+u_i)^{-1}, \tag{16}$$

$$\frac{\partial ll((u_1,x_1),...,(u_n,x_n))}{\partial \beta_j} = \sum_{i=1}^{n} \frac{1}{w(x_i,t_{l,i}+u_i)} \frac{\partial w(x_i,t_{l,i}+u_i)}{\partial \beta_j} + \left(\frac{k}{\tau(t_{l,i}+u_i)^{-1}} - t_i\right)\frac{\partial \tau(t_{l,i}+u_i)^{-1}}{\partial \beta_j}, \tag{17}$$

*In this research we ignore the ongoing (censored) interevent time at the end of the catalog since its influence was negligible. More generally, the log-likelihood for a process with censored times equals $\sum_{i=1}^{n} -H(u_i) + \log(h(u_i)) \cdot \delta_i$, where δ_i represents the event indicator for observation i (for censored times $\delta_i = 0$).

where

$$\frac{\partial\tau(t)^{-1}}{\partial\beta_j} = \frac{1}{15}\sum_{x=1}^{15}\left[y_j(x,t)\exp\left(\sum_{j=1}^{J}\beta_j y_j(x,t)\right)\right], \tag{18}$$

$$\frac{\partial w(x_i,t)}{\partial\beta_j} = w(x_i,u_i)\sum_{x=1}^{15}\left[\left(y_j(t,x_i)-y_j(x,t)\right)w(x,t)\right], \tag{19}$$

and $\psi(x) = \frac{d}{dx}\log(\Gamma(x))$ equals the Digamma function. Moreover, the Hessian of Eq. (14) is equal to

$$\frac{\partial ll((u_1,x_1),...,(u_n,x_n))}{\partial k^2} = -n\frac{\partial}{\partial k^2}\log(\Gamma(k)), \tag{20}$$

$$\frac{\partial ll((u_1,x_1),...,(u_n,x_n))}{\partial k\partial\tau_0} = n, \tag{21}$$

$$\frac{\partial ll((u_1,x_1),...,(u_n,x_n))}{\partial k\partial\beta_j} = \sum_{i=1}^{n}\frac{1}{\tau(t_{l,i}+u_i)^{-1}}\frac{\partial\tau(t_{l,i}+u_i)^{-1}}{\partial\beta_j}, \tag{22}$$

$$\frac{\partial ll((u_1,x_1),...,(u_n,x_n))}{\partial\tau_0\partial\beta_j} = \sum_{i=1}^{n} -u_i\frac{\partial\tau(t_{l,i}+u_i)^{-1}}{\partial\beta_j}, \tag{23}$$

$$\begin{aligned}\frac{\partial ll((u_1,x_1),...,(u_n,x_n))}{\partial\beta_{j_1}\partial\beta_{j_2}} =& \sum_{i=1}^{n}\Bigg[\sum_{x=1}^{15}\left[\left(y_j(t_{l,i}+u_i,x_i)-y_j(t_{l,i}+u_i,x)\right)\tfrac{\partial w(x,t_{l,i}+u_i)}{\partial\beta_{j_2}}\right] \\ &+\left(\tfrac{k}{\tau(t_{l,i}+u_i)^{-1}}-u_i\right)\tfrac{\partial\tau(t_{l,i}+u_i)^{-1}}{\partial\beta_{j_1}\partial\beta_{j_2}}+\left(\tfrac{k}{\tau(t_{l,i}+u_i)^{-2}}\tfrac{\partial\tau(t_{l,i}+u_i)^{-1}}{\partial\beta_{j_1}}\tfrac{\partial\tau(t_{l,i}+u_i)^{-1}}{\partial\beta_{j_2}}\right)\Bigg],\end{aligned} \tag{24}$$

where

$$\frac{\partial\tau(t)^{-1}}{\partial\beta_j} = \frac{1}{15}\sum_{x=1}^{15}\left[y_{j_1}(x,t)y_{j_2}(x,t)\exp\left(\sum_{j=1}^{J}\beta_j y_j(x,t)\right)\right]. \tag{25}$$

Our optimization strategy is as follows. We numerically find the roots of the gradient (here the Hessian is used). These parameter values are now used as the starting values to numerically (Broyden-Fletcher-Goldfarb-Shanno algorithm (4) is used) find the maximum-likelihood estimates for the complex model.

2. Determination of model covariates and parameter values for Groningen

In this study we have excluded events from the KNMI catalog based on a magnitude of completeness equal to 1.3 as suggested in (5).

2.1. Candidate covariates Groningen. The covariates taken into account in the case-study analysis can be found in Table S1. The stationary covariates are derived from the geological *top Rotliegend surface model* from the NAM provided via TNO (6). For the compaction covariates we use the compaction model provided by Shell (7).

Covariate	Definition	Scaled by	Standardized by
$y_{\dot{C}}(x,t)$	Average compaction (m/day) per region.	10^5	0.77
$y_C(x,t)$	Average cumulated compaction (m), per region at the time of previous tremor.	10	1.47
$y_F(x)$	Sum of total length (m) of faults in (x,y)-plane.	10^{-5}	0.82
$y_D(x)$	Weighted (by length between observation points) average of the dip values (degrees) from the NAM *top Rotliegend surface model*.	1	78.65
$y_S(x)$	% total length of faults having a strike $\in (45,90)$ degrees north-east in the (x,y)-plane.	1	0.41
$y_T(x)$	Weighted (by length between observation points) average of the throw values (m) from the NAM *top Rotliegend surface model*.	10^{-1}	0.65
$y_R(x)$	Weighted (by length between observation points) average throw-thickness ratio.	1	0.33

Table S1. Definition model covariates for Groningen model.

For stability of the numerical optimization of the likelihood the covariates are transformed. First the $y_F(x)$ is scaled by 10^{-5}, $y_T(x)$ by 10^{-1}, $y_{\dot{C}}(x,t)$ by 10^5 and $y_C(x,t)$ by 10. Subsequently, all covariates are standardized by their mean (over the regions, see Table S1), e.g. $y_C(x,t) := 10y_C(x,t) - 1.47$. The numerical values of the stationary covariates obtained by this procedure can be found in Table S2. The values of the time-varying covariates per region are graphically presented in Fig. S1.

region	1	2	3	4	5	6	7	8	9	10	11	12	13	14	15
y_F	-0.63	0.14	0.23	0.25	0.28	0.26	0.43	0.28	-0.17	0.38	0.12	-0.30	-0.34	-0.26	-0.67
y_D	2.06	0.44	-3.42	-1.52	1.95	3.10	-0.16	0.88	-1.40	-0.84	-1.64	1.88	-1.64	-1.71	2.02
y_S	-0.01	0.00	-0.12	-0.08	0.05	-0.03	0.12	-0.03	0.13	0.07	0.02	0.13	0.06	-0.10	-0.24
y_T	-0.10	0.33	0.01	0.36	-0.02	0.08	-0.21	-0.18	0.52	-0.09	-0.28	-0.31	0.17	-0.05	-0.23
y_R	-0.07	0.25	0.14	0.57	-0.08	0.01	-0.09	-0.02	0.10	-0.11	-0.17	-0.17	-0.06	-0.12	-0.18

Table S2. Stationary covariates in each region.

Furthermore, we considered a critical total compaction by introducing a truncation c, mimicking the compaction level at which all faults in a region have reached their fault strength. The Gamma time-scale parameter used in the Groningen case study equals

$$\tau(t) = \tau_0 15 \left(\sum_{x=1}^{15} \exp\left(\beta_C \min\{c, y_C(x,t)\} + \sum_{j=2}^{J} \beta_j y_j(x,t) \right) \right)^{-1}. \tag{26}$$

2.2. Final model. In statistics it is common to rely on a forward- or backward-selection procedure to select covariates that should be included in the model. However, the optimal model can easily be missed when covariates are highly correlated. We therefore fit all possible models including an effect of the total compaction (since this is the only covariate that can explain the increase in intensity over time). Each model is fit using the approach explained in Section 1.3. However, the derivative of $ll((u_1,x_1),...,(u_n,x_n))$ with respect to the compaction-truncation variable c does not exist. The gradient can be derived given a value of c. For several values of the c variable we numerically find the roots of the gradient (here the Hessian is used). Subsequently, the different c fits are compared and the parameters giving rise to the highest log-likelihood are selected. In turn these estimates are used as the starting values of the numerical optimization.

The final model is selected based on Akaike's information criteria (AIC) (8) to prevent overfitting. Below we present the (log-likelihood-based) best-fitting model with J covariates involved.

It is important to notice that the k estimate proves insensitive to the model choice, thus our estimate for the fraction of triggered events is insensitive to the model choice. Based on the AIC the model with covariates total compaction, compaction rate, total fault density, percentage of faults with specific strike angle, throw-thickness ratio and truncation is selected. The effect estimates and corresponding standard errors are presented in Table S4.

The final estimates define the development of $\tau(t)$, which evolution is displayed in Fig. S2. This background rate, $1/\tau(t)$, is partitioned over the 15 regions using the weights of the Gamma IAFT model. For a subset of the regions, the evolution of the proportion of seismic events over time is displayed in Fig. S3.

The effect estimates and cap estimate presented in Table S4 can be transformed back such that they can be interpreted in the units of the original covariates introduced in Table S1, e.g. the cap variable in meters compaction is estimated as $\frac{0.94+1.47}{10}$ with a corresponding standard error of $\frac{0.044}{10}$, and are presented in Table 1 of the main text. The final model thus includes a

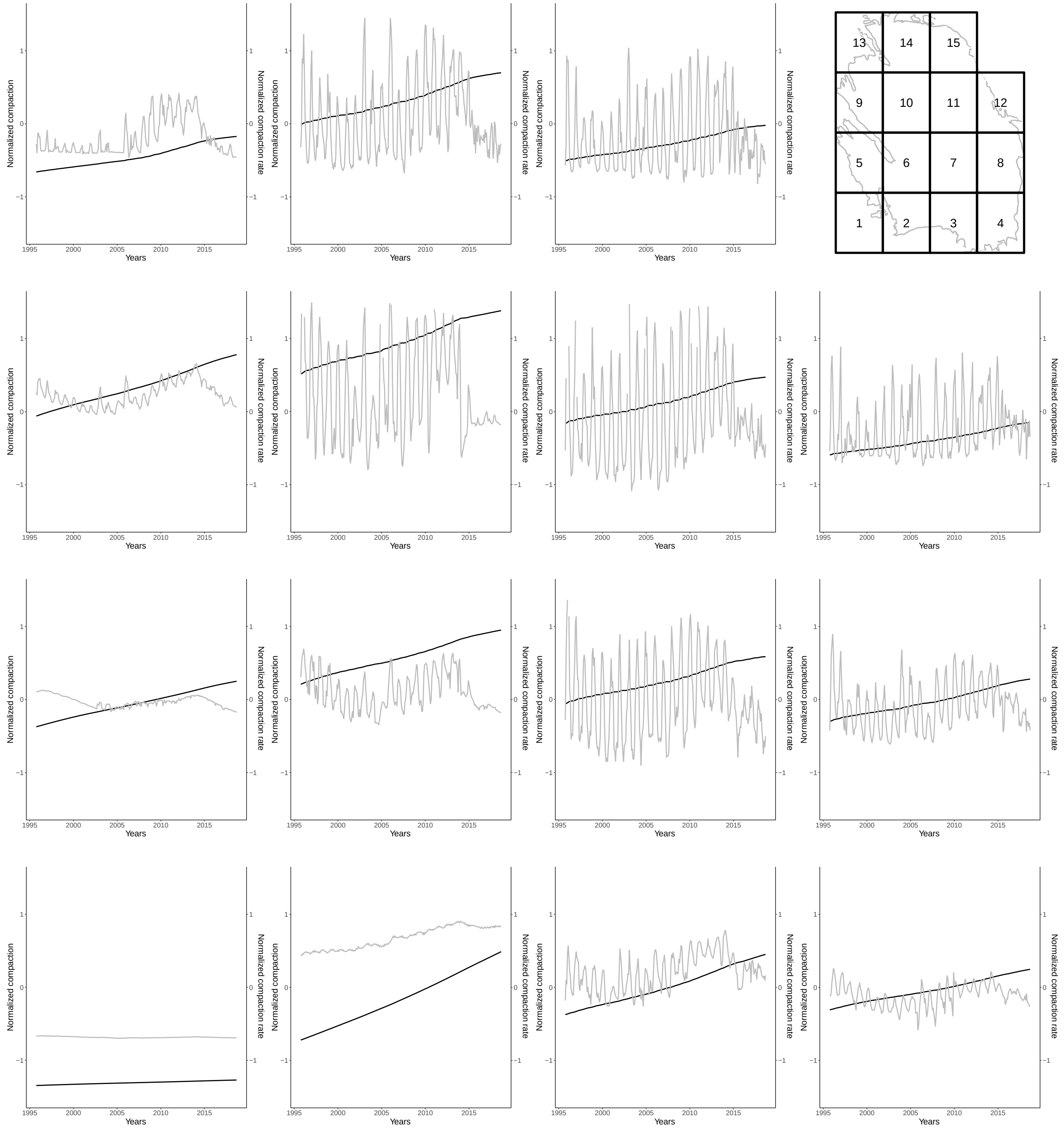

Fig. S1. Change of $y_C(x,t)$ (solid black) and $y_{\dot{C}}(x,t)$ (solid grey) over time per region. Graphs are positioned following the geometry of the regions over the field (right upper corner), e.g. $y_{\dot{C}}(13,t)$ and $y_C(13,t)$ are shown in the left upper corner.

k	$\log(\tau_0)$	$\beta_{\dot{C}}$	β_F	β_D	β_S	β_T	β_R	β_C	$\beta_{C*\dot{C}}$	c	$-ll$	AIC	J	BIC
0.71	-4.14	0	0	0	0	0	0	2.39	0	-	2373.20	4752.41	3	4764.35
0.71	-4.14	0	0	0	0	0	0	2.39	0	-	2373.20	4752.41	3	4764.35
0.71	-4.17	0	0	0	3.89	0	0	2.21	0	-	2357.26	4722.53	4	4738.45
0.72	-4.29	0	0	0	4	0	0	2.64	0	1	2346.83	4703.66	5	4723.56
0.72	-4.31	0	0	0	4.37	-0.80	0	2.71	0	0.94	2340.72	4693.43	6	4717.32
0.73	-4.31	0.26	0	0	4.36	-0.88	0	2.64	0	0.94	2337.66	4689.32	7	4717.19
0.73	-4.32	0.26	0.63	0	3.44	0	-1.44	2.38	0	0.94	2335.80	4687.60	8	4719.45
0.73	-4.33	0.37	0.62	0	3.46	0	-1.50	2.39	-0.18	0.94	2335.58	4689.15	9	4724.98
0.73	-4.34	0.37	0.78	0	3.18	0.30	-1.95	2.33	-0.17	0.94	2335.48	4690.96	10	4730.77
0.73	-4.34	0.37	0.74	0.01	3.28	0.26	-1.91	2.34	-0.17	0.94	2335.46	4692.91	11	4736.71

Table S3. Maximum-likelihood Gamma IAFT-model parameter estimates using $J = 3, ..., 10$ covariates respectively.

k	$\log(\tau_0)$	$\beta_{\dot{C}}$	β_F	β_S	β_R	β_C	c
0.73 (0.031)	4.32 (0.059)	0.26 (0.095)	0.63 (0.164)	3.44 (0.621)	-1.44 (0.422)	2.38 (0.084)	0.94 (0.044)

Table S4. Final Gamma IAFT-model parameter estimates and corresponding standard errors.

truncation on the total compaction. This effect is shown for each region in Fig. S4. The Gamma IAFT model suggests that all faults in the most active region 10, the Loppersum area, were critically stressed at the end of 2007, when the local compaction was 0.24 meter.

2.2.1. Risk of bias due to successive-event overlaps in seismograms. In real catalogs, short interevent times cannot be detected because waveforms of successive-event overlaps in seismograms. As a result it is impossible to observe short interevent times below some threshold. The observed interevent times are thus realization of a conditional distribution (random interevent time $U > u_0$, for some threshold u_0). If the probability that $U \leq u_0$ is reasonably large, then this conditional distribution seriously deviates from the full distribution. Parameter estimates obtained by fitting the non-conditional distribution will then be biased. Instead one can fit the conditional distribution by maximizing

$$\begin{aligned} \tilde{ll}((u_1, x_1), ..., (u_n, x_n)) &= \log\left(\prod_{i=1}^{n} f_i(u_i, x_i)/S_i(u_0)\right) \\ &= \log\left(\prod_{i=1}^{n} f_i(u_i, x_i)\right) - \sum_{i=1}^{n} \log S_i(u_0) \\ &= ll((u_1, x_1), ..., (u_n, x_n)) + \sum_{i=1}^{n} H_i(u_0), \end{aligned} \tag{27}$$

where f_i, S_i, H_i are the pdf, survival function and integrated hazard function of the ith interevent-time distribution.

The potential bias is the result of the $\sum_{i=1}^{n} H_i(u_0)$ contribution that lowers minus the log-likelihood. It is thus important to evaluate $\sum_{i=1}^{n} H_i(u_0)$ after the model has been fitted. If the contribution is low, then the conditional distribution is close to the full distribution and the thresholding will not result in bias of the estimates.

In the Groningen catalog, which we used to fit the final model (Tables S3 and S4), the smallest interevent time was equal to 0.00069 days (1 minute). From Groningen seismograms, see e.g. (9, 10), it becomes clear that the time between the first-P-wave arrival at a seismic station and the moment when the signal amplitude has decayed below the first-P-wave amplitude is in the order of seconds. Thus, it is reasonable to assume that all interevent times larger than or equal to 1 minute are observed. Using the model fitted in this study we find that $\sum_{i=1}^{397} H_i(0.00069) = 0.2$, which is extremely small compared to the final minus log-likelihood of 2335.8. In this case we thus do not suffer from time-threshold bias. In the case the thresholded distribution does deviate from the unconditional distribution one should maximize Eq. (27) instead of Eq. (13). As an example we present the thresholded-distribution fit using $u_0 = 5$ minutes, such that we fit 395 interevent times, which estimates are presented in Table S5. Indeed the estimates are similar to those presented in Table S4.

k	$\log(\tau_0)$	$\beta_{\dot{C}}$	β_F	β_S	β_R	β_C	u_0
0.73 (0.032)	4.32 (0.059)	0.27 (0.094)	0.64 (0.164)	3.45 (0.622)	-1.41 (0.422)	2.38 (0.084)	0.94 (0.044)

Table S5. Final Gamma IAFT model-parameter estimates using the thresholded $u_0 = 5$ minutes distribution.

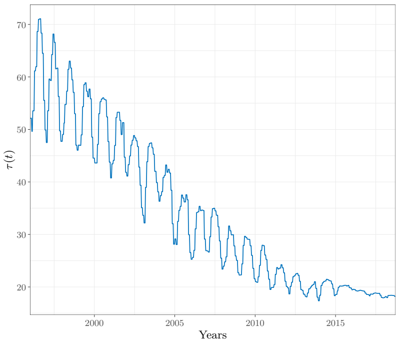

Fig. S2. Evolution of $\tau(t)$, see Eq. (26), over time.

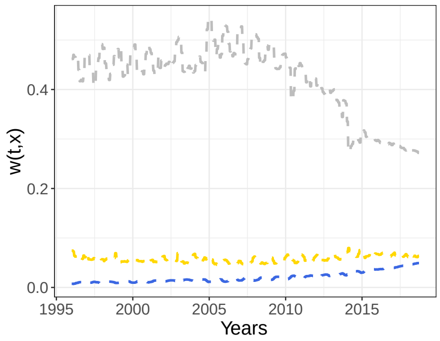

Fig. S3. Evolution of $w(10,t)$ (grey), $w(2,t)$ (yellow) and $w(11,t)$ (blue) over time.

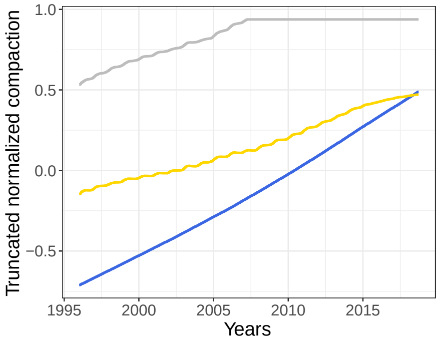

Fig. S4. Evolution of $\min\{10y_C(x,t) - 1.47, 0.94\}$ for x=10 (grey), x=2 (yellow) and x=11 (blue) over time.

2.3. Comparison with the memoryless IAFT model.

To test the presence of earthquake-earthquake interaction we compare the final Gamma IAFT-model fit with the fit of an Exponential ($k = 1$)-model. The maximum-likelihood estimates of the parameters of the Exponential model equal:

k	$\log(\tau_0)$	$\beta_{\dot{C}}$	β_F	β_S	β_R	β_C	c
1	4.02 (0.059)	0.29 (0.100)	0.61 (0.178)	3.41 (0.695)	-1.44 (0.481)	2.40 (0.088)	0.94 (0.049)

Table S6. Exponential IAFT-model parameter estimates based on Groningen catalog 1995-2018.

Based on the likelihood-ratio test ($p < 0.0001$) we reject the hypothesis $H_0 : k = 1$ and conclude that the improved fit cannot be explained by chance alone. It is important to note that both models are fitted to the Groningen dataset and thus roughly expected the same number of events in the period October 1st 1995 - October 1st 2018. The difference between the models is clearly illustrated by plotting the temporal hazards over time, see Fig. S5.

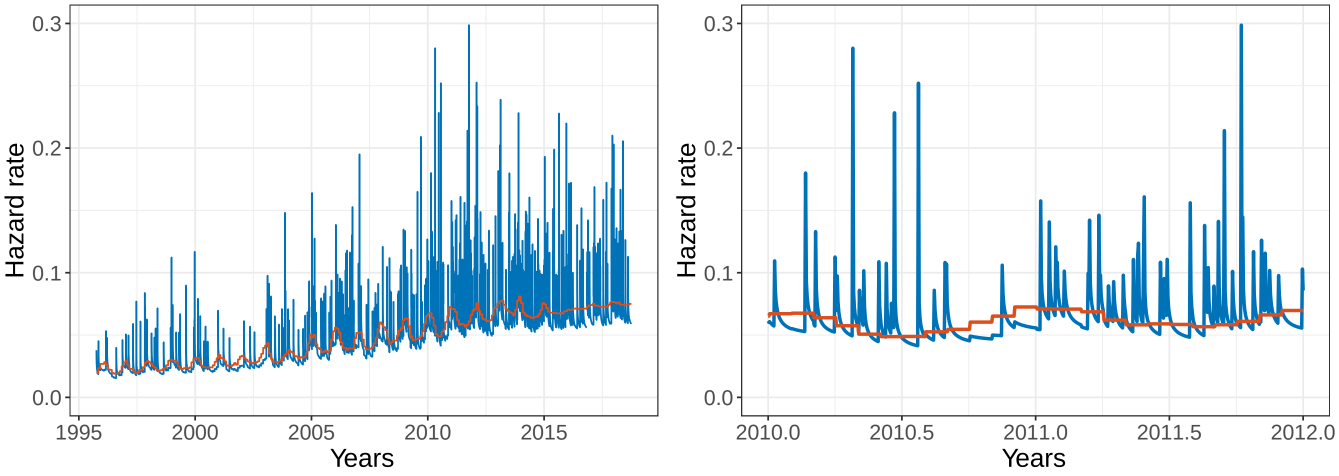

Fig. S5. Change of hazard for the Gamma IAFT model (blue) and the Exponential IAFT model (red) in the period 1995-2018 (left) and 2010-2012 (right).

The hazard of the Gamma IAFT model is far more spiky than the hazard of the Exponential IAFT model. The Gamma IAFT model results in a more clustered profile of events as illustrated in Fig. 3a.

3. Model validation with simulated Groningen data

The final model obtained in Section 2.2 for the Groningen field results in an estimate of the non-stationary driving function $1/\tau(t)$ and an interevent-time distribution after each event time t_l. Since in the Groningen case the time scales of variation of τ vs. t and h vs. u turn out to be well separated, the instantaneous distribution, following each t_l, can be approximated by a Gamma distribution. As a result the time variation of τ can be scaled out, giving a single Gamma, $\Gamma(\hat{k}, 1)$, distribution vs. the variable $U/\tau(t_l + U)$ over the full time period. This function is shown in Fig. S6 together with the actual rescaled, by $\hat{\tau}(t)$, Groningen interevent data. The pdf of $\frac{U}{\hat{\tau}(t_l+U)}$ is estimated as the bin proportions, $\hat{p}$, divided by the width of the

bin. The confidence intervals are obtained by deriving the confidence intervals for the bin proportions ($\hat{p} \pm 1.96 \frac{\sqrt{\hat{p}(1-\hat{p})}}{\sqrt{396}}$) and subsequently dividing both bounds by the width of the bin. The $\frac{u}{\hat{\tau}(t_l+u)}$ observations are randomly distributed over time

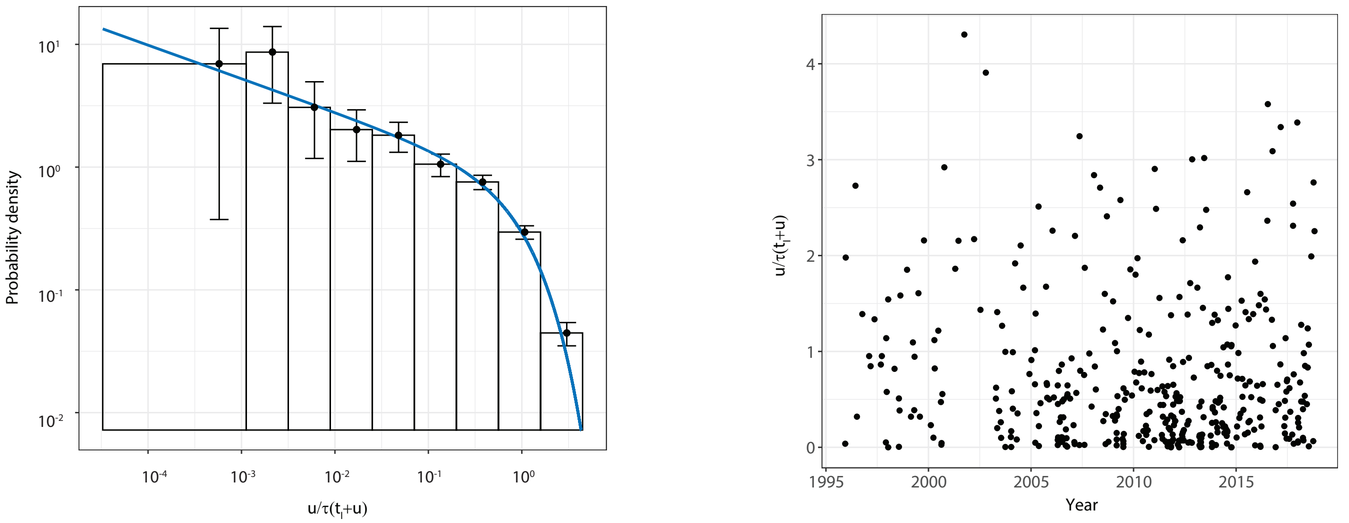

Fig. S6. Empirical probability-density estimates for $\frac{U}{\hat{\tau}(t_l+U)}$ (left, black dots), with pointwise 95% confidence intervals, and the $\Gamma(\hat{k}, 1)$ pdf (left, blue). The logarithmic bins used for the density-estimates are shown and the breaks equal $(0, 3.3 \cdot 10^{-5}, 1.1 \cdot 10^{-3}, 3.2 \cdot 10^{-3}, 8.9 \cdot 10^{-3}, 0.03, 0.07, 0.2, 0.6, 1.6, 4.5)$. The number of observations per bin equals $(3, 7, 7, 13, 33, 54, 109, 120, 51)$ respectively. Furthermore, $\frac{u}{\hat{\tau}(t_l+u)}$ have been plotted over time (right).

(Wald-Wolfowitz runs test, $p = 0.17$), see Fig. S6. Thus, once we account for the change in intensity over the years the mixed distribution of background and triggered interevent times can be well described by this universal Gamma curve. However, since the data are scarce, the agreement between curve and data is in itself no sufficient validation of our model, and more formal validation tests will be presented below.

3.1. Simulation description. To simulate past-event catalogs based on the final model we rely on the theory of survival analysis. In general a random variable with cumulative distribution function (cdf) F is generated by drawing a realization of a Uniform $[0, 1]$ random variable V and evaluating the inverse cdf at this V, $F^{-1}(V)$. Equivalently, one could evaluate the survival function, $S = 1 - F$, at the random V. From survival analysis we know $F(u) = 1 - \exp(-H(u))$. Our Gamma IAFT model defines the hazard rate, and thus the integrated hazard $H(u)$, presented in Eq. (11), and the cdf.

The maximum-likelihood estimates of the final model theoretically follow an asymptotic multivariate normal distribution[†], see e.g. (11). More precisely

$$\sqrt{n}\left(\hat{\boldsymbol{\theta}}_n - \boldsymbol{\theta}\right) \xrightarrow{d} \mathcal{N}(0, I_{\theta}^{-1}), \tag{28}$$

where $\hat{\boldsymbol{\theta}}_n$ are the maximum-likelihood estimates for $(k, \beta_1, ..., \beta_J)$ based on a sample of size n, $\boldsymbol{\theta} = (k, \beta_1, ..., \beta_J)$, $\xrightarrow{d}$ represents convergence in distribution and I_θ equals the Fisher information matrix. Let $\hat{\boldsymbol{\theta}}$ represent the maximum-likelihood estimates of the Gamma IAFT-model parameters. We estimate the total variance $\hat{\Sigma}$, by computing (numerically) the inverse of the Hessian of the log-likelihood evaluated at the maximum-likelihood estimates $\hat{\boldsymbol{\theta}}$. The first step in the simulation of an earthquake catalog consists of drawing a realization $\boldsymbol{\theta}$ of the multivariate normal $\mathcal{N}\left(\hat{\boldsymbol{\theta}}, \hat{\Sigma}\right)$.

Subsequently, a clock is set to $t = 0$ (October 1995 in the Groningen case study) and a interevent time U_1 is realized. Furthermore, a multinomial random variable, with probabilities equal to the Gamma IAFT-model weights at time U_1, is realized to determine the event location. Next, the clock is set to U_1, after which a second interevent time, U_2, is realized based on the integrated hazard after U_1 and the location of this event is generated based on the weights at time $U_1 + U_2$. These steps continue until N events are realized such that $\sum_{i=1}^{N} U_i$ exceeds the length of the period of interest. For the Groningen case study the total number of days between September 1st 1995 and October 1st 2018 equals 8431.

Thousand generated catalogs based on the models fitted to the interevent times from the Groningen catalog (Gamma IAFT and Exponential IAFT) are shown in Fig. S7.

[†] This theory holds for the model presented as Eq. (10) and Eq. (12). However, for model Eq. (26) used in the Groningen case, $\boldsymbol{\theta} = (k, c, \beta_1, ..., \beta_J)$ and the theory breaks down since $c \mapsto \min\{c, y_C(x,t)\}$ is not differentiable w.r.t to c at $y_C(x,t)$. In practice the parameter estimates will not change if we use a smooth approximation of the min function. Using this approximation the theory does apply and thus allows us to rely on the discussed result by numerically deriving the inverse of the Hessian of the log-likelihood evaluated at the maximum-likelihood estimates $\hat{\boldsymbol{\theta}}$.

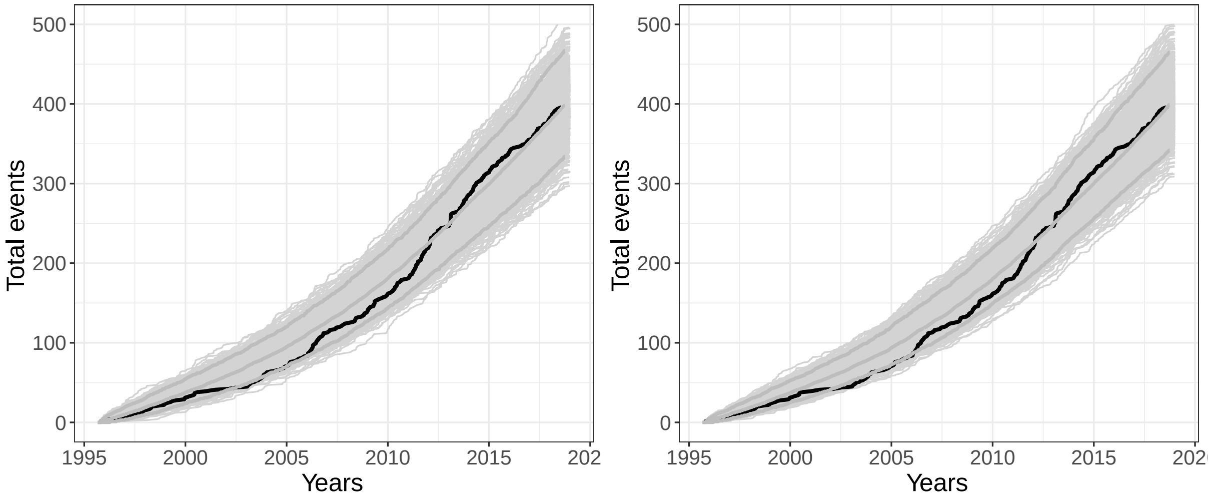

Fig. S7. Observed Groningen catalog (black) and thousand simulated catalogs (light grey), Gamma IAFT model (left) and Exponential IAFT model (right). Furthermore, the 2.5%, 50.0%, and 97.5% pointwise quantiles of the simulations are presented (grey).

3.2. Formal tests.

3.2.1. Cox-Snell residuals. A quantitative way of validating an interevent-time model makes use of the integrated hazard at the event times, $H(U_1), H(U_2), ..., H(U_N)$. In the context of assessing the validity of a survivor function these integrated hazards at the event times are referred to as *Cox-Snell* residuals. If the hazard is accurately modeled, then the *Cox-Snell* residuals should follow a unit Exponential distribution, see e.g. (12). This can be proven using the survival-analysis equivalence relation presented as Eq. (2):

$$\begin{aligned}\mathbb{P}\left(H(U) > x\right) &= \mathbb{P}\left(-\log(S(U)) > x\right) \\ &= \mathbb{P}\left(U > S^{-1}(\exp(-x))\right) \\ &= S\left(S^{-1}(\exp(-x))\right) \\ &= \exp(-x)\ \square.\end{aligned}$$

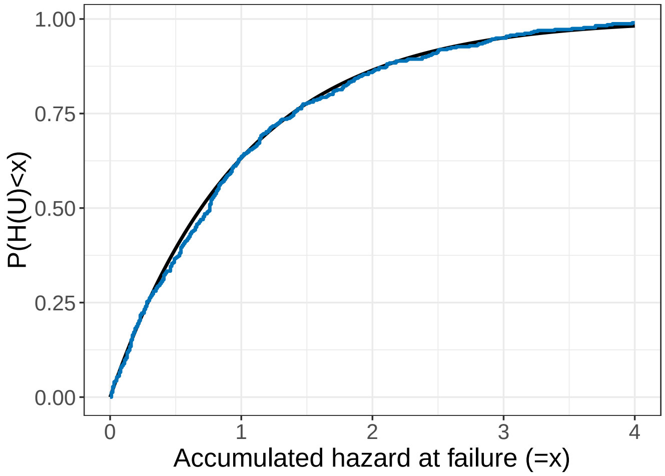

Fig. S8. Empirical cdf of Cox-Snell residuals for the Gamma IAFT model (blue) and the cdf of a unit Exponential distribution (black).

The empirical distribution of the *Cox-Snell* residuals can be compared to the unit Exponential distribution using the Kolmogorov-Smirnov (KS) test (13). For the final model the p-value of this test is equal to 0.681, from which we conclude that the proposed Gamma IAFT model seems suitable. The p-value of the KS test comparing the unit Exponential with the *Cox-Snell* residuals of the Exponential IAFT model instead equals 0.009, as a result of the underestimation of the clustering. This explains the superiority of our final IAFT model compared to the Exponential IAFT model once more.

3.2.2. Randomness. The distribution of the *Cox-Snell* residuals can be used to test for overall performance. However, in non-stationary processes it is very important to investigate the randomness in the sequence of the residuals. E.g. in a setting where the *Cox-Snell* residuals are expected to be smaller at the beginning of the catalog and larger towards the end, the proposed model does not describe the underlying mechanism of the data. To test whether the *Cox-Snell* residuals (sorted on event date) are independent and identically distributed we apply Wald-Wolfowitz runs test (13). The resulting p-value equals 0.159, from which we conclude that there is no reason to doubt the appropriateness of the model.

3.2.3. Spatial fit. We have stressed that appropriate modeling of the spatial heterogeneities is key to model the temporal clustering. To validate the spatial fit we use Pearson's chi-squared test (13). More specifically, the test statistic equals

$$\chi^2 = \sum_{i=1}^{15} \frac{(x_i - \mathbb{E}_{\text{Gamma IAFT}}[X_i])^2}{\mathbb{E}_{\text{Gamma IAFT}}[X_i]}.$$

The expected counts, $\mathbb{E}_{\text{Gamma IAFT}}[X_i]$, per region are estimated from the thousand simulations and presented in Fig. S9 (left). For the Groningen data the statistic equals $\chi^2 = 37.8$ and the contributions per region are presented in Fig. S9 (right).

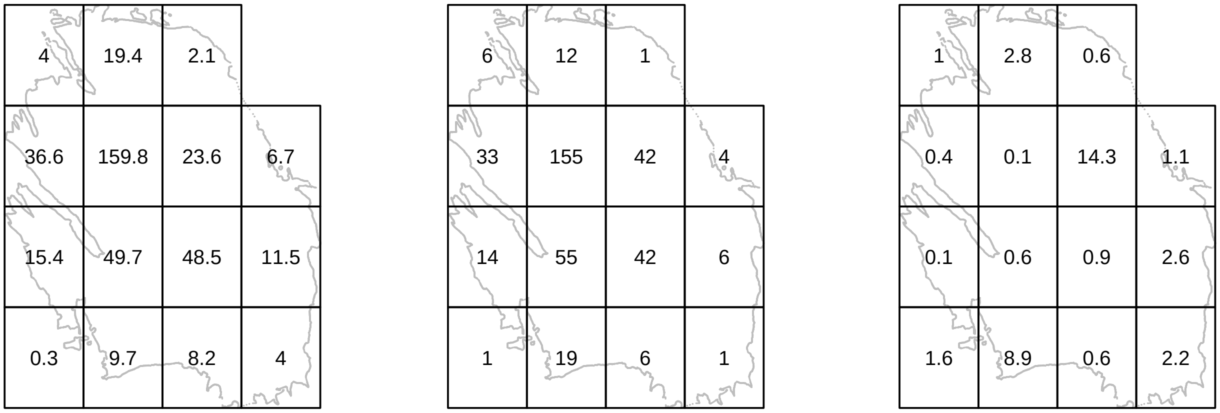

Fig. S9. Spatial fit: $\mathbb{E}_{\text{Gamma IAFT}}[X_i]$ (left), observations (mid) and the contribution to χ^2 per region (right).

The distribution of χ^2 assuming the Gamma IAFT model is approximated using the thousand simulations. Based on this empirical distribution we find

$$\mathbb{P}(\chi^2 > \chi^2_{\text{data}}) = 0.048.$$

This probability is rather low and thus questions the spatial fit of our model. However, with a closer look at the expected and realized counts in Fig. S9, it becomes clear that the counts in region 11 are deviating from the expected counts. Several of the events in region 11 are close to the boundary of region 10 and the other way around, see Fig. S10. A slight change in number of events allocated to region 10 or 11 could result in a different goodness-of-fit value. Merging the two regions results in a

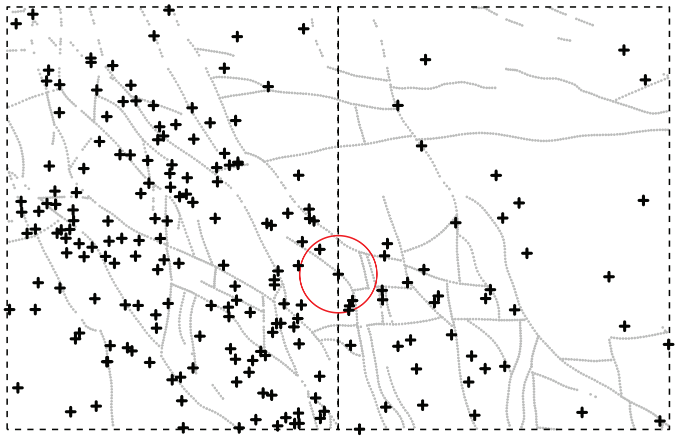

Fig. S10. Observed events in region 10 and 11 and their uncertainty region (5) (red, 1 km radius).

chi-square statistic of $\widetilde{\chi^2} = 24.5$, with a p-value of

$$\mathbb{P}(\widetilde{\chi^2} > \widetilde{\chi^2}_{\text{data}}) = 0.24.$$

This shows that our model is capable of reproducing the spatial distribution over the field.

3.3. Hindcast.

3.3.1. Gamma IAFT model. Finally, we investigate how well we can forecast the number of events in the period 2014-2018 based on the information available before that period. This is of particular interest since the production of gas has drastically decreased from this period on, giving rise to compaction rates that have not been observed before. When we want to interpret our claims causally we should be able to predict event counts in a production scenario that has not been observed so far. The maximum-likelihood estimates of the Gamma IAFT model based on the period 1995-2013 are given in Table S7. Simulations

k	$\log(\tau_0)$	$\beta_{\dot{C}}$	β_F	β_S	β_R	β_C	c
0.71 (0.035)	4.33 (0.071)	0.21 (0.102)	0.20 (0.193)	3.76 (0.739)	-1.09 (0.504)	2.66 (0.104)	0.91 (0.048)

Table S7. Gamma IAFT-model parameter estimates (and corresponding standard errors) based on Groningen catalog 1995-2013.

for the 2014-2018 period of the Gamma IAFT model with the parameters from Table S7 are presented in Fig. S11.

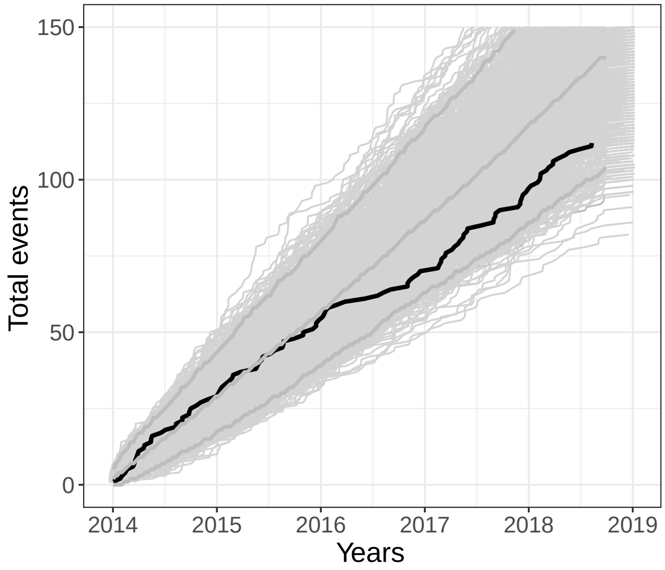

Fig. S11. Hindcast Gamma IAFT model (light grey) and the observed Groningen catalog from 2014-2018. Furthermore, the 2.5%, 50.0%, and 97.5% pointwise quantiles of the simulations are presented (grey).

3.3.2. Ignoring spatial heterogeneity. The importance of taking care of the spatial heterogeneity can be illustrated by studying the hindcast with a model with no spatial (ns) heterogeneity and only describing the temporal process. Now, we model the scale parameter of the Gamma IAFT model, τ_{ns}, as

$$\tau_{\text{ns}}(t) = \tau_0 \exp\left(\beta_{C(t)} \min\left(c, \sum_{x=1}^{15} y_C(x,t)\right) + \beta_{\dot{C}(t)} \left(\sum_{i=1}^{15} y_{\dot{C}}(x,t)\right)\right)^{-1}. \tag{29}$$

The maximum-likelihood estimates of this temporal model based on the period $1995 - 2013$ can be found in Table S8. In this case, the compaction truncation did not improve the model fit. The simulations based on this model can be found in Fig. S12

k	$\log(\tau_0)$	$\beta_{\dot{C}}$	β_C	c
0.72 (0.036)	3.39 (0.070)	0.38 (0.197)	3.56 (0.475)	-

Table S8. Model parameter estimates, when spatial heterogeneity is ignored, based on the 1995-2013 catalog.

and clearly are a bad predictor of the evolution of the real Groningen catalog in the period 2014-2018. This illustrates the importance of modeling the spatial dissimilarities even if the focus is on the temporal evolution.

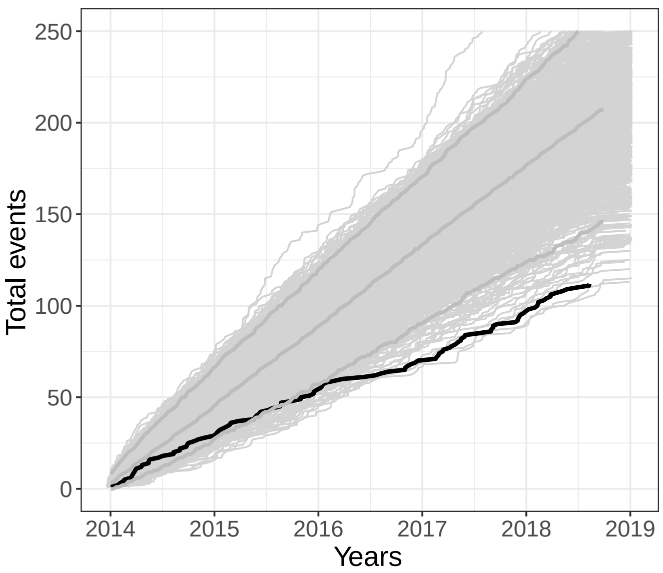

Fig. S12. Hindcast model ignoring spatial heterogeneity (light grey) and the observed Groningen catalog from 2014-2018. Furthermore, the 2.5%, 50.0%, and 97.5% pointwise quantiles of the simulations are presented (grey).

4. Catalog declustering and aftershock fraction

4.1. Stochastic declustering. An earthquake process can be viewed as the union of the operator-triggered process with a process triggered by the earthquake-earthquake interaction. The random interevent time U thus equals $\min(U_0, U_1)$, where U_0 and U_1 equal the interevent times in the absence of aftershocks and background events respectively.

Lemma 1. *Let U_0 and U_1 be independent random variables of which the distributions are defined by the hazard functions $h_0(u)$ and $h_1(u)$ respectively. Then, the hazard function of $U = \min(U_0, U_1)$ equals $h_0(u) + h_1(u)$*

Proof. Using equivalence relation Eq. (2) we find

$$\begin{aligned} S(u) &= \mathbb{P}(\min(U_0, U_1) > u) \\ &= \mathbb{P}(U_0 > u)\mathbb{P}(U_1 > u) \\ &= \exp\left(-\int_{t=0}^{u} h_0(t)dt\right) \exp\left(-\int_{0}^{t=u} h_1(t)dt\right) \\ &= \exp\left(-\int_{t=0}^{u} \left(h_0(t) + h_1(t)\right) dt\right). \end{aligned}$$

Thus $H(u) = \int_{t=0}^{u} (h_0(t) + h_1(t))\, dt$ and $h(u) = h_0(t) + h_1(t)$. □

The Gamma IAFT model can be used to stochastically decluster an earthquake catalog by labeling events as a triggered or background event based on random (Bernoulli) experiments.

Theorem 2. *The probability of an event to originate from the background process, given the interevent time u, equals*

$$p_{background}(u) = \frac{h_{k=1}(u)}{h(u)}, \tag{30}$$

where $h_{k=1}(u) = \frac{1}{\tau(t_l+u)}$ and equals the Gamma IAFT hazard at u when setting k equal to 1.

Proof. Let $U = \min(U_0, U_1)$, be the random interevent time starting at t_l where U_0 and U_1 equal the times to the next background event and to the next triggered, by earthquake-earthquake interaction, event respectively. Here U_0 is the interevent time, after t_l of an inhomogeneous Poisson process with (background) rate $1/\tau(t_l + u)$. Using Bayes‘ theorem and the

survival-analysis equivalence relation Eq. (4), given $U = u$, we find

$$\begin{aligned}
\mathbb{P}(U_1 > u \mid U = u) &= \frac{f(U = u \mid U_1 > u)\mathbb{P}(U_1 > u)}{f(U = u)} \\
&= \frac{f(U_0 = u \mid U_1 > u)\mathbb{P}(U_1 > u)}{f(U = u)} \\
&= \frac{f(U_0 = u)\mathbb{P}(U_1 > u)}{f(U = u)} \\
&= \frac{f(U_0 = u)S_1(u)}{f(U = u)} \\
&= \frac{S_0(u)h_0(u)S_1(u)}{f(U = u)} \\
&= \frac{S_0(u)h_0(u)S_1(u)}{h(u)S(u)} \\
&= \frac{S_0(u)h_0(u)S_1(u)}{h(u)S_0(u)S_1(u)} \\
&= \frac{h_0(u)}{h(u)}.
\end{aligned}$$

Applying Lemma 1 results in $\mathbb{P}(U_1 > u \mid U = u) = \frac{h_0(u)}{h_0(u)+h_1(u)}$ and substituting $h_0(u) = 1/\tau(t_l + u)$ gives the result. □

By drawing a Uniform random variable, V, for each event we label the event as a background event when $V < p_{\text{background}}(u_i)$ and as a triggered event otherwise. To decluster the catalog of Groningen we start by drawing Gamma IAFT-model parameter realizations from the multivariate normal distribution discussed in Section 3.1 and start labeling each event (background or triggered) using $p_{\text{background}}(u_i)$ given $\boldsymbol{\theta}_j$. Some examples of stochastically declustered catalogs of Groningen can be found in Fig. S13. At the end of the catalog (October 2018) the declustered catalogs contain on average 73% of the events.

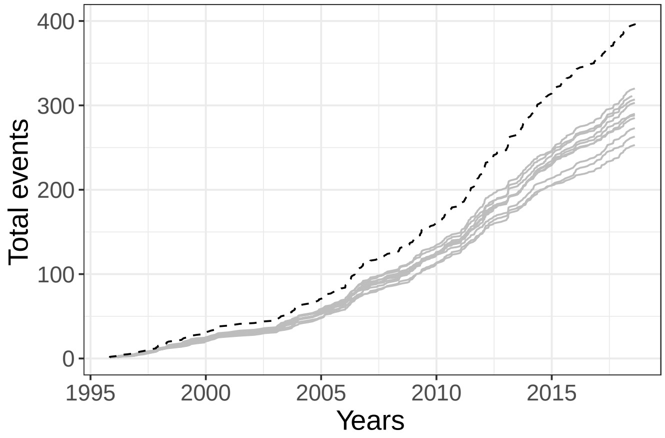

Fig. S13. Ten stochastically declustered Groningen catalogs based on the Gamma IAFT model (grey) and as a reference the original catalog (dashed black).

4.2. Fraction of background events. We would like to compute the % of background events in the Gamma IAFT model. To do so we are interested in the expected value of the background-event probability, the ratio between the background rate and the total hazard of the Gamma IAFT model, at interevent time U (after t_l). The complex interevent-time pdf can be derived using the survival-analysis equivalence relations

$$f(u) = S(u) \cdot h(u) = S(u) \cdot \tilde{h}(u) = \frac{S(u)}{\tilde{S}(u)} \tilde{f}(u), \tag{31}$$

where $\tilde{h}(u)$, $\tilde{f}(u)$ and $\tilde{S}(u)$ equal respectively the hazard function, pdf and survival function of the stationary Gamma distribution with scale parameter $\tau(t_l + u)$. Since the pdf and cdf have no elegant closed form expression it is hard to derive $\mathbb{E}\left[\frac{h_0(U)}{h_U}\right]$. Let us assume there are a finite number of intensity changes over the period of interest. Now, we can define the n intensity changes at time $s_1, s_2, ..., s_n$ after t_l. During the interval $[t_l + s_i, t_l + s_{i+1})$ the background rate is constant and can

be referred to as $\frac{1}{\tau_i}$. After time $t_l + s_n$, the background rate stays constant at the level of $\frac{1}{\tau_{n+1}}$. Without loss of generality we assume $s_0 = 0$. Then the expected proportion of background events can be computed as

$$\begin{aligned}
\mathbb{E}\left[p_{\text{background}}(U)\right] &= \mathbb{E}\left[\frac{\tau(t_l+u)^{-1}}{\frac{\tilde{f}(U)}{\tilde{S}(U)}}\right] \\
&= \int_0^\infty \tau(t_l+s)^{-1} S(s)ds \\
&= \sum_{i=1}^{n} \tau_i^{-1} \int_{s_{i-1}}^{s_i} S(s)ds + \tau_{n+1}^{-1}\int_{s_n}^{\infty} S(s)ds \\
&= \sum_{i=1}^{n}\left[\left(\prod_{j=1}^{i-1}\frac{\Gamma(k,\frac{s_j}{\tau_j})}{\Gamma(k,\frac{s_{j-1}}{\tau_j})}\right)\frac{\int_{s_{i-1}}^{s_i}\frac{1}{\tau_i}\Gamma(k,\frac{t}{\tau_i})dt}{\Gamma(k,\frac{s_{i-1}}{\tau_i})}\right] + \left(\prod_{j=1}^{n}\frac{\Gamma(k,\frac{s_j}{\tau_j})}{\Gamma(k,\frac{s_{j-1}}{\tau_j})}\right)\frac{\int_{s_n}^{\infty}\frac{1}{\tau_{n+1}}\Gamma(k,\frac{t}{\tau_{n+1}})dt}{\Gamma(k,\frac{s_n}{\tau_{n+1}})} \\
&= k - \frac{\Gamma(k+1,\frac{s_1}{\tau_1}) + \frac{\Gamma(k,\frac{s_1}{\tau_1})}{\Gamma(k,\frac{s_1}{\tau_2})}\Gamma(k+1,\frac{s_1}{\tau_2}) - \sum_{i=2}^{n}\left(\prod_{j=1}^{i-1}\frac{\Gamma(k,\frac{s_j}{\tau_j})}{\Gamma(k,\frac{s_j}{\tau_{j+1}})}\right)\cdot\left(\Gamma(1+k,\frac{s_i}{\tau_i}) - \frac{\Gamma(k,\frac{s_i}{\tau_i})}{\Gamma(k,\frac{s_i}{\tau_{i+1}})}\Gamma(1+k,\frac{s_i}{\tau_{i+1}})\right)}{\Gamma(k)}.
\end{aligned} \tag{32}$$

If the scale parameter $\tau(t)$ does not change during each interevent period, then each interevent time is Gamma distributed, with shape parameter k and a scale parameter that might differ per interevent time, and the expression simplifies to $\frac{\Gamma(k+1)}{\Gamma(k)} = k$. If the scale parameter $\tau(t)$ does change, $\mathbb{E}\left[p_{\text{background}}(U)\right]$ will differ from k, but can be derived via simulation. For each simulation $\frac{1}{n_j}\sum_{i=1}^{n_j} p_{\text{background}}(u_i)$ can be computed and by the Central Limit Theorem, the average over all simulations will converge to $\mathbb{E}\left[p_{\text{background}}(U)\right]$. For the Gamma IAFT model using the maximum-likelihood estimates, derived from the Groningen catalog, this fraction equals 0.73 and indeed equals k. This is a consequence of the differences between $1/\tau_i$ and $1/\tau_{i+1}$ being small and thus the complex distributions are very comparable to (constant scale-parameter) Gamma distributions.

4.3. Confidence interval of the fraction of background events in Groningen. In an induced-seismicity study, like Groningen, the stochastic process underlying the data can be modeled. The observed data can then be viewed as one specific realization of the stochastic model. Note that the fraction of background events can differ per realization of the stochastic model and can deviate from the expected fraction of the process (the latter has been discussed in the previous section). For the real data the event times are known and the fraction should thus be estimated conditioned on these event times. The ith event has a probability equal to $p_{\text{background}}(u_i)$ to be a background event, thus the expected number of background events is equal to $\sum_{i=1}^{n} p_{\text{background}}(u_i)$. For a Gamma IAFT model with the maximum-likelihood estimates, the expected number of background events is equal to the expected number of the stochastic process, $\mathbb{E}\left[\sum_{i=1}^{n} p_{\text{background}}(U_i)\right]$, discussed in the previous section, and thus equals 290 (73% of the total).

Each event label, $L_i = \mathbb{1}_{\text{event } i \text{ is a background event}}$, is Bernoulli distributed with the just-defined background probability. Therefore, the number of background events, $N = \sum_{i=1}^{n} L_i$, follows a so-called *Poisson binomial* distribution with parameters $p_1, p_2, ..., p_n$ (14). Confidence limits of the number of background events in a realized catalog of a known Gamma IAFT model are thus equal to the quantiles of this Poisson binomial distribution. In practice the parameters of the Gamma IAFT model are unknown and therefore estimated, which introduces uncertainty. Let $\boldsymbol{\theta}$ represent the real Gamma IAFT model parameters, then the distribution of $N \mid \boldsymbol{\theta}$ is the Poisson binomial distribution just introduced. Now, let $\boldsymbol{\theta}$ itself be a multivariate random variable with law $f_{\boldsymbol{\theta}}$. Then, the distribution of N equals

$$\mathbb{P}(N = n) = \int_{\boldsymbol{\theta}\in\boldsymbol{\Theta}} \mathbb{P}(N = n \mid \boldsymbol{\theta}) f_{\boldsymbol{\theta}} d\boldsymbol{\theta}, \tag{33}$$

where $\boldsymbol{\Theta}$ is the support of the $\boldsymbol{\theta}$ variable, namely $\{k > 0, \tau_0 > 0, \beta_C \in \mathbb{R}, ...\}$. Let us assume that $\boldsymbol{\theta}$ follows the asymptotic multivariate normal distribution of the maximum-likelihood estimate (of the Gamma IAFT model) discussed in Section 3.1. Since it is analytically very difficult to compute the 2.5% and 97.5% quantiles we have used a Monte Carlo approach to derive the distribution of N. For $j = 1$ to $j = 10000$:

1. We have generated $\boldsymbol{\theta}_j$ from $\mathcal{N}\left(\hat{\boldsymbol{\theta}}, \hat{\Sigma}\right)$.

2. We have generated N_j from the Poisson binomial distribution with $p_{\text{background}}(u_i)$.

Finally, the 2.5% and 97.5% quantiles of the empirical distribution are computed. For the Groningen catalog ($n = 397$), we have concluded that the confidence interval (CI) for the number of background events equals $(256, 324)$, which equals $(64.5\%, 81.6\%)$ of all events. [‡] Note that the CI of k, based on the asymptotic normal distribution of $\hat{k}$, equals $(66.8\%, 79.2\%)$ and is thus tighter.

[‡] To verify that the empirical distribution represents the distribution of N we check that the CI based on only the first five thousand simulated catalogs, $(255, 325)$, is similar to the CI based on all simulations.

5. Comparison with ETAS-based models and data

The Epidemic-Type Aftershock-Sequence (ETAS) model is often used in practice to analyze seismicity (15). In the ETAS model the occurrence rate of aftershocks at time t due to an event at time v decreases according to the modified Omori law $\frac{K}{(c+t-v)^{1+\theta}}$, where K, c and θ are model parameters (16). Each event of magnitude m triggers aftershocks with a rate proportional to $10^{\alpha(m-m_{min})}$, where m_{min} is the magnitude of completeness. The hazard of the temporal ETAS model equals

$$h_{\text{ETAS}}(t) = 1/\tau(t) + \sum_{i=1}^{n(t)} \frac{K}{(c+t-v_i)^{1+\theta}} 10^{\alpha(m_i - m_{min})}. \tag{34}$$

Here $1/\tau(t)$ represents the background rate as in the Gamma IAFT model, $n(t)$ equals the total number of past events before time t, v_i and m_i represent the event time and magnitude of the ith event. The ratio of background-to-total events can, only in steady-state, be directly related to the so-called branching ratio, g, the number of first-generation daughter events per background event (17). For $\theta > 0$ and $\alpha < b$, we obtain that $g = \frac{K}{c^\theta} \frac{b}{b-\alpha} \frac{1}{\theta}$, where b is the parameter of the Gutenberg-Richter distribution. The integrated hazard of the ETAS model equals

$$H_{\text{ETAS}}(t) = \int_{s=0}^{t} 1/\tau(s) ds + \frac{K}{\theta} \sum_{i=1}^{n(t)} 10^{\alpha(m_i - m_{min})} \left(c^{-\theta} - (c+t-v_i)^{-\theta}\right). \tag{35}$$

The integrated hazard defines the survivor function $S_{\text{ETAS}}(t)$, which allows us to simulate with the ETAS model, following the same lines as explained in Section 3.1.

5.1. Groningen-based ETAS model. In this study our focus is on the Gamma IAFT model rather than an ETAS model, since the small data base and the complexity of a non-stationary driving force asks for a parsimonious parameter set to avoid arbitrariness. We want to show that this parameter-poor Gamma model can nevertheless be effectively used on a Groningen catalog simulated with a temporal ETAS model. Based on the Cox-Snell test we assume that the τ_0 and $\boldsymbol{\beta}$ estimates obtained from the Gamma IAFT fit to the Groningen catalogs are accurately describing the background rate. To find values for the parameters c, θ, α and g that give rise to synthetic catalogs that are representative of the real Groningen catalog we evaluate the log-likelihood

$$ll_{\text{ETAS}}(v_1, ..., v_n \mid c, \alpha, \theta, g, \tau_0, \boldsymbol{\beta}) = \sum_{i=1}^{n} -H_{\text{ETAS}}(v_i) + \log(h_{\text{ETAS}}(v_i)), \tag{36}$$

where $n = 396$, over a grid of candidate values. The grid consists of $c \in \{10 \text{ min}, 1 \text{ hour}, 6 \text{ hours}, 12 \text{ hours}, 1 \text{ day}\}$, $\alpha \in \{0, 0.1, 0.3, 0.5, 0.7, 0.9\}$, $\theta \in \{0.05, 0.10, 0.15, 0.20\}$ and $g \in \{0.1, 0.2, 0.3, 0.4, 0.5, 0.6, 0.7, 0.8, 0.9\}$ (cf. (16)). In Table S9, per c value, the parameter sets that give rise to the two best fits and the corresponding log-likelihood values are presented. Accurate estimation of the four remaining ETAS parameters requires a sufficiently large number of observations. To illustrate that in the Groningen catalog this is not the case, we compared the two best fits, $(c = 6h, \alpha = 0.3, \theta = 0.05, g = 0.8)$ and $(c = 6h, \alpha = 0.3, \theta = 0.10, g = 0.4)$. Their log-likelihoods are insignificantly different and indicate a strong intercorrelation of θ and g. The resulting systems are however vastly different in their cross-over times for saturation of individual aftershock sequences (17), and hence in intersequence overlap. Consistent with the choice of the Gamma timescale $\tau(t)$ as the true input background rate, we selected the fit with $g = 0.4$, i.e., limited intersequence overlap (18).

$c[day]$	α	θ	g	ll_{ETAS}
0.25	0.3	0.05	0.8	-1533.977
0.25	0.3	0.10	0.4	-1534.240
0.5	0.3	0.05	0.4	-1534.951
0.5	0.3	0.15	0.4	-1534.953
0.042	0.3	0.05	0.4	-1535.862
0.042	0.5	0.05	0.4	-1536.364
1	0.3	0.15	0.4	-1535.921
1	0.3	0.20	0.4	-1536.190
0.0069	0.3	0.05	0.4	-1540.633
0.0069	0.5	0.05	0.4	–1541.017

Table S9. Log-likelihood of ETAS model, given $\tau_0, \boldsymbol{\beta}$, evaluated at different values for the ETAS parameters. In this table per c value the best two fits are presented.

Synthetic ETAS-simulated catalogs mimicking the Groningen data are then obtained with $c = 6$ hours, $\theta = 0.10$, $\alpha = 0.3$ and $g = 0.4$ see Fig. S14. Based on 500 simulations our temporal ETAS model gives rise to a mean fraction of 76.6% background events over the course of the Groningen catalog, see Fig. S16a. (Indeed this does not equal $1 - g$, as the system is not in steady state.) On a par with the Gamma IAFT model the temporal ETAS model did not consider individual event locations, although differences in compaction behavior over the Groningen field were accounted for via the used time-varying background rate.

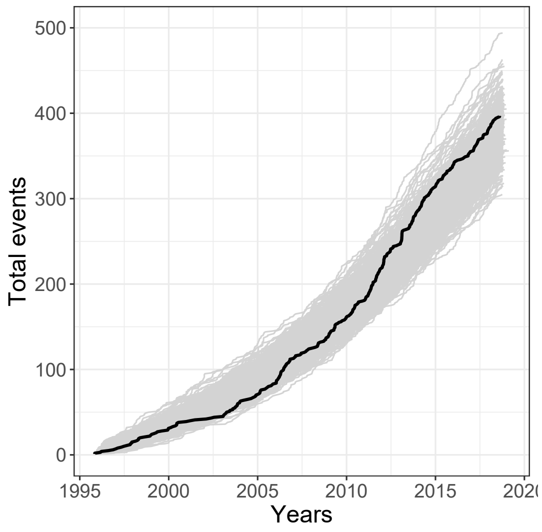

Fig. S14. Observed Groningen (solid black) catalog and non-stationary ETAS-simulated catalogs (solid grey), $n_{\text{sim}} = 500$.

5.2. Gamma IAFT-model fit to ETAS simulations. To show the appropriateness of the Gamma IAFT model we first fitted it, following Section 1.3, to the union of all interevent times from all 500 simulations, by optimizing the log-likelihood:

$$ll_{\text{IAFT}}(u_{1,1}, ..., u_{1,n_1}, u_{2,1}, ..., u_{500,n_{500}} \mid k, \boldsymbol{\beta}) = \sum_{j=1}^{500} \sum_{i=1}^{n_j} -H(u_i) + \log(h(u_i)), \tag{37}$$

which gives in particular $k = 0.746$. Following the same procedure as in the paper and Section 3.2.1, we have compared the cdf of the Cox-Snell residuals from this Gamma IAFT-model fit, considering its maximum-likelihood estimates, to the cdf of a unit Exponential, see Fig. 3a (main text) and Fig. S15. We conclude that the interevent times generated by the temporal ETAS model are indeed well approximated by the Gamma IAFT-model distribution.

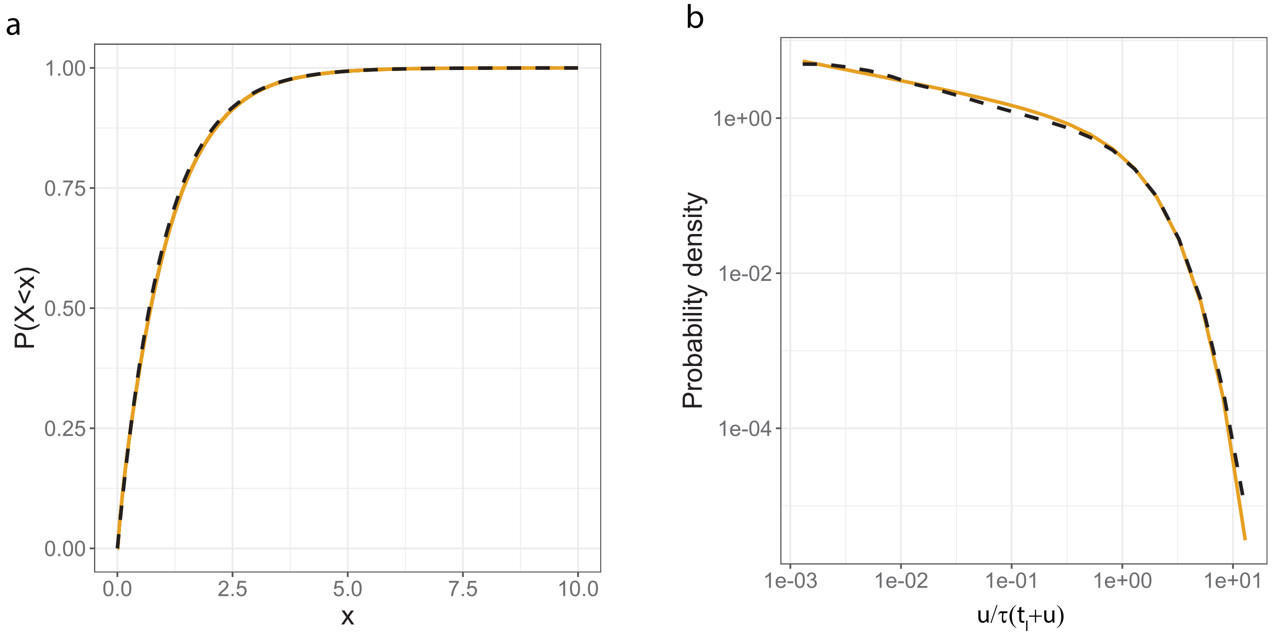

Fig. S15. (**a**), Empirical cdf of the Cox-Snell residuals from the Gamma IAFT fit to the union of the ETAS-simulated catalogs (solid yellow), compared with the cdf of a unit Exponential (dashed black). (**b**), The pdf of $\frac{U}{\tau(t_l+U)}$ using a logarthmic binning of events (dashed black), compared with the pdf $\Gamma(0.746, 1)$ (solid yellow).

5.3. Background fraction estimation based on Gamma IAFT model or ETAS model. As the fraction of aftershocks is a stochastic quantity (19), the 'true' expected background fraction, $\mu_{BF}(g,\theta,c,\boldsymbol{\beta})$, differs per synthetic catalog and equals $n^{-1}\sum_{i=1}^{n}\left[\frac{\tau(v_i)^{-1}}{h_{\text{ETAS}}(v_i)}\right]$, see Fig. S16a. We would like to investigate the performance of the estimation of the expected background fraction by fitting the ETAS model or the Gamma IAFT model to the synthetic ETAS catalogs.

To do so, we have fitted the Gamma IAFT model to each synthetic ETAS catalog, by derivation of the estimates $\hat{k},\hat{\tau}_0$ and $\hat{\boldsymbol{\beta}}$ that maximize the log-likelihood

$$ll_{\text{IAFT}}(u_1,...,u_n \mid k,\tau_0,\boldsymbol{\beta}) = \sum_{i=1}^{n} -H(u_i) + \log(h(u_i)). \tag{38}$$

Similarly, we have fitted the ETAS model (as defined by Eq. (34) and Eq. (35)) to each single synthetic ETAS catalog, by derivation of the estimates $\hat{g},\hat{\alpha},\hat{\theta},\hat{c},\hat{\tau}_0$ and $\hat{\boldsymbol{\beta}}$ that maximize the log-likelihood Eq. (36).

Based on these maximum-likelihood estimates of the Gamma IAFT fit and the ETAS fit we have calculated, for each synthetic ETAS catalog at each event time the background rate $\hat{\tau}(t_l+u)$ and the hazard rate $\hat{h}(t_l+u)$ or $\hat{h}_{\text{ETAS}}(v)$. Subsequently, the fraction of background events was estimated as $n^{-1}\sum_{i=1}^{n}\left[\frac{\hat{\tau}(v_i)^{-1}}{\hat{h}(u_i)}\right]$ and $n^{-1}\sum_{i=1}^{n}\left[\frac{\hat{\tau}(v_i)^{-1}}{\hat{h}_{\text{ETAS}}(v_i)}\right]$ per synthetic ETAS catalog.In summary, for 500 synthetic catalogs:

**Synthetic ETAS catalog fitting**
1a: Simulate synthetic ETAS catalog with $(c=6h,\alpha=0.3,\theta=0.10,g=0.4)$.
1b: Compute expected background fraction $\mu_{BF}(c=6h,\alpha=0.3,\theta=0.10,g=0.4,\boldsymbol{\beta})$.
2a: Fit Gamma IAFT model.
2b: Estimate expected background fraction using $\hat{k},\hat{\tau}_0,\hat{\boldsymbol{\beta}}$.
3a: Fit ETAS model.
3b: Estimate expected background fraction using $\hat{c},\hat{\alpha},\hat{\theta},\hat{g},\hat{\tau}_0,\hat{\boldsymbol{\beta}}$.

5.3.1. Efficiency. For each synthetic catalog, we have compared the estimated background fractions to $\mu_{BF}(g,\theta,c,\boldsymbol{\beta})$. A histogram of the difference is presented as Fig. S16b. The median of these errors equals 0.03 and -0.03 for the ETAS and the Gamma IAFT fit respectively. We conclude that, despite the finite sample size, the background rate and % of background events are appropriately estimated with the Gamma IAFT model.

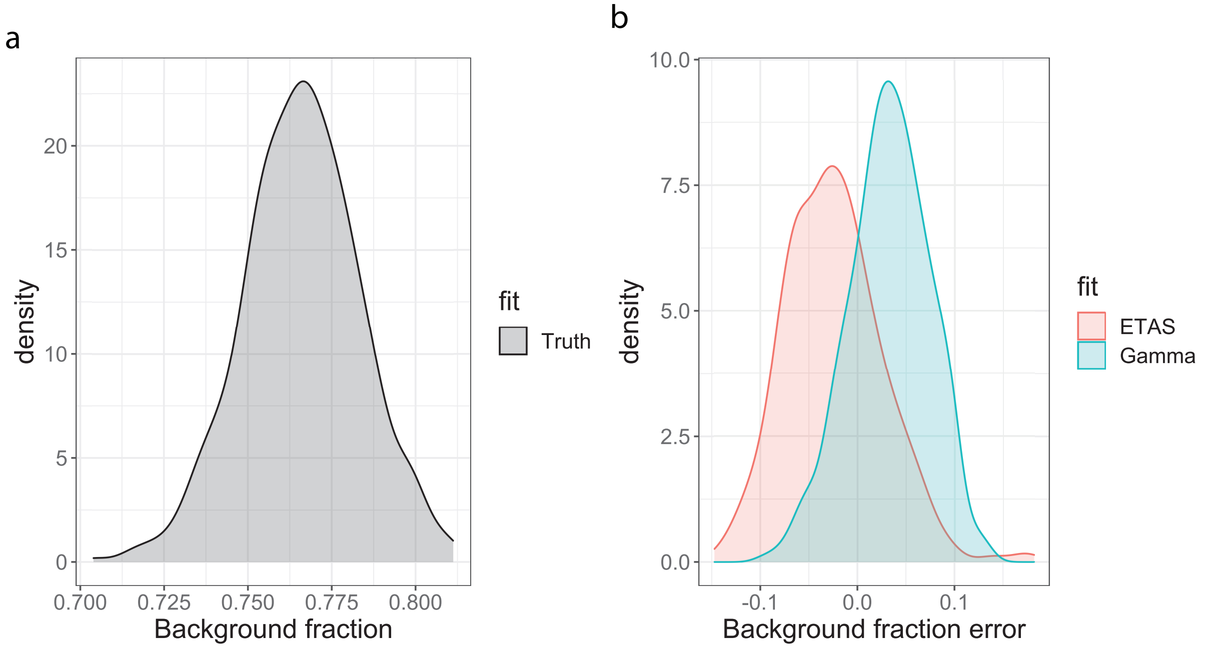

Fig. S16. (**a**) Expected background-fraction distribution over the 500 synthetic ETAS catalogs. (**b**), Difference between real expected fraction of background events and the estimates based on the Gamma IAFT-model fit (blue) and the ETAS-model fit (red) respectively, using the ETAS-simulated catalogs, $n_{\text{sim}} = 500$.

The variances of these background-fraction estimates are referred to as σ^2_{ETAS} and σ^2_{IAFT}. We find $\hat{\sigma}^2_{\text{ETAS}} = 0.05$ and $\hat{\sigma}^2_{\text{IAFT}} = 0.04$ respectively. The standard error is significantly higher when fitting an ETAS model (Brown-Forsythe test, $H_0 : \sigma^2_{\text{ETAS}} \leq \sigma^2_{\text{IAFT}}$, $p < 10^{-4}$). Hence, even for the synthetic Groningen-based ETAS catalogs, the fraction of triggered events can be estimated more efficiently by fitting a Gamma IAFT model.

5.4. Power of the Cox-Snell test for validating Gamma behavior. Actual catalogs as well as synthetic ETAS catalogs do not always conform to Gamma interevent distributions (20). To show how sensitively our approach can validate the appropriateness of the Gamma distribution we simulated 1000 times 397 events for different stationary ETAS models. We used $\tau/c = 10^5$, setting the values of n and θ such that, depending on the branching ratio and the tail of the Omori function, both catalogs far from and close to the asymptotic steady state were obtained; the base case $n = 0.9$, $\theta = 0.03$, which is still far from steady state, corresponds to Fig. 1 in (20). The settings give rise to distributions that deviate from Gamma behavior, see Fig. S17. Consequently, the (Gamma) maximum-likelihood estimators of the shape (k) and scale (τ) parameters deviate from the real fraction of background events and the inverse background rate respectively. In these settings the maximum-likelihood estimator of the shape parameter, $\hat{k}$, should thus not be interpreted as the fraction of background events.

setting	n	θ	% background	$\hat{k}$	$\hat{\tau}$
A	0.9	0.03	65.8 %	0.372	52.90
B	0.9	0.16	19.3 %	0.164	34.81
C	0.9	0.20	16.1 %	0.150	31.69
D	0.61	0.03	76.7 %	0.455	50.58
E	0.3	0.03	88.5 %	0.620	42.80

Table S10. Real fraction of background events, Gamma $\hat{k}$ and $\hat{\tau}$ per ETAS setting.

setting	n	θ	$\alpha = 0.05$	$\alpha = 0.10$
A	0.9	0.03	100 %	100 %
B	0.9	0.16	100 %	100 %
C	0.9	0.20	100 %	100 %
D	0.61	0.03	88.4 %	94.3 %
E	0.3	0.03	79.0 %	84.2 %

Table S11. Power Gamma goodness-of-fit test using significance level α.

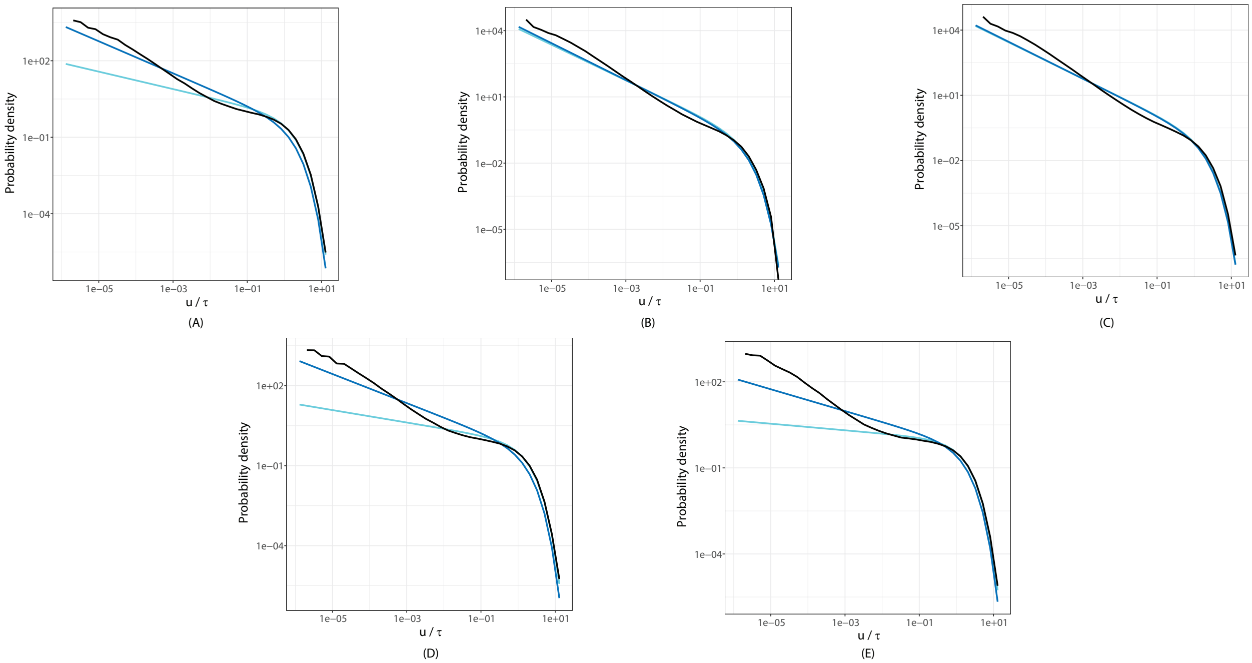

Fig. S17. Empirical estimate of the pdf for $\frac{U}{\tau}$, using a logarithmic binning of all $(1000 \cdot 396)$ simulated interevent times with the ETAS model (black line), the $\Gamma($ % background, $1)$ pdf (cyan) and the $\Gamma(\hat{k}, 1)$ pdf (blue) for settings A-E described in Table S10.

Nevertheless, as shown in Table S11, the power (probability of rejecting the null-hypothesis) of the goodness-of-fit test (of the Gamma distribution), based on the Cox-Snell residuals discussed in 3.2.1, is very high for these scenarios. So the Cox-Snell test can sensitively verify the appropriateness of the Gamma model. The interpretation of the Gamma IAFT model presented in this research is only valid after such formal model validation.

References

1. PJ Smith, *Analysis of Failure and Survival Data.* (CRC Press, Boca Raton, FL), (2017).
2. DY Lin, Z Ying, Semiparametric inference for the accelerated life model with time-dependent covariates. *J. Stat. Plan. Inference* **44**, 47–63 (1995).
3. B Efron, *Large-scale inference: Empirical Bayes methods for estimation, testing, and prediction.* (Cambridge University Press), pp. 1–263 (2012).
4. CG Broyden, The convergence of a class of double-rank minimization algorithms: 2. The new algorithm. *IMA J. Appl. Math. (Institute Math. Its Appl.* **6**, 222–231 (1970).

5. B Dost, E Ruigrok, J Spetzler, Development of seismicity and probabilistic hazard assessment for the Groningen gas field. *Geol. en Mijnbouw/Netherlands J. Geosci.* **96**, s235–s245 (2017).
6. Nederlandse Aardolie Maatschappij, Technical Addendum to the Winningsplan Groningen 2016 - Part 1: Summary & Production, (Nederlandse Aardolie Maatschappij B.V., Assen), Technical Report April (2016).
7. S Bierman, D Randell, M Jones, No. SR.17.01246, (Shell Global Solutions International B.V., Amsterdam), Technical report (2017).
8. H Akaike, A New Look at the Statistical Model Identification. *IEEE Trans. Autom. Contr.* **19**, 716–723 (1974).
9. J Spetzler, B Dost, Hypocentre estimation of induced earthquakes in Groningen. *Geophys. J. Int.* **209**, 453–465 (2017).
10. B Paap, D Kraaijpoel, M Bakker, HN Gharti, Wave propagation modelling of induced earthquakes at the Groningen gas production site. *Geophys. J. Int.* **214**, 1947–1960 (2018).
11. AW van der Vaart, *Asymptotic Statistics.* (Cambridge University Press, Cambridge), (1998).
12. Y Ogata, Statistical models for earthquake occurrences and residual analysis for point processes. *J. Am. Stat. Assoc.* **83**, 9–27 (1988).
13. DJ Sheskin, *Handbook of Parametric and Nonparametric Statistical Procedures.* (CRC Press, Boca Raton, FL) No. 3, (2004).
14. LJ Gleser, On the Distribution of the Number of Successes in Independent Trials. *Ann. Probab.* **3**, 182–188 (1975).
15. Y Ogata, Space-time point-process models for earthquake occurrences. *Ann. Inst. Stat. Math.* **50**, 379–402 (1998).
16. S Hainzl, F Scherbaum, C Beauval, Estimating background activity based on interevent-time distribution. *Bull. Seism. Soc. Am.* **96**, 313–320 (2006).
17. A Helmstetter, D Sornette, Subcritical and supercritical regimes in epidemic models of earthquake aftershocks. *J. Geophys. Res. Solid Earth* **107**, ESE 10–1–ESE 10–21 (2002).
18. S Touati, M Naylor, IG Main, M Christie, Masking of earthquake triggering behavior by a high background rate and implications for epidemic-type aftershock sequence inversions. *J. Geophys. Res. Solid Earth* **116** (2011).
19. A Saichev, A Helmstetter, D Sornette, Power-law distributions of offspring and generation numbers in branching models of earthquake triggering. *Pure Appl. Geophys.* **162**, 1113–1134 (2005).
20. A Saichev, D Sornette, "Universal" distribution of interearthquake times explained. *Phys. Rev. Lett.* **97**, 078501 (2006).
